# The Impact of COVID-19 on Weak-Form Efficiency in Cryptocurrency and Forex Markets

**DOI:** 10.3390/e25121622

**Published:** 2023-12-05

**Authors:** Pavlos I. Zitis, Shinji Kakinaka, Ken Umeno, Stavros G. Stavrinides, Michael P. Hanias, Stelios M. Potirakis

**Affiliations:** 1 Department of Electrical and Electronics Engineering, University of West Attica, Ancient Olive Grove Campus, Egaleo, 12241 Athens, Greece; pzitis@uniwa.gr; 2 Department of Applied Mathematics and Physics, Graduate School of Informatics, Kyoto University, Sakyo, Kyoto 606-8501, Japan; kakinaka.shinji.d74@kyoto-u.jp (S.K.); umeno.ken.8z@kyoto-u.ac.jp (K.U.); 3 Department of Physics, International Hellenic University, 65404 Kavala, Greece; s.stavrinides@ihu.edu.gr (S.G.S.); mhanias@physics.ihu.gr (M.P.H.); 4 National Observatory of Athens, Metaxa and Vasileos Pavlou, Institute for Astronomy, Astrophysics, Space Applications and Remote Sensing, Penteli, 15236 Athens, Greece; 5 Department of Electrical Engineering, Computer Engineering and Informatics, School of Engineering, Frederick University, Nicosia 1036, Cyprus

**Keywords:** market efficiency, COVID-19, Bitcoin, cryptocurrencies, forex market, complexity, entropy, multifractal analysis, complex systems, econophysics

## Abstract

The COVID-19 pandemic has had an unprecedented impact on the global economy and financial markets. In this article, we explore the impact of the pandemic on the weak-form efficiency of the cryptocurrency and forex markets by conducting a comprehensive comparative analysis of the two markets. To estimate the weak-form of market efficiency, we utilize the asymmetric market deficiency measure (MDM) derived using the asymmetric multifractal detrended fluctuation analysis (A-MF-DFA) approach, along with fuzzy entropy, Tsallis entropy, and Fisher information. Initially, we analyze the temporal evolution of these four measures using overlapping sliding windows. Subsequently, we assess both the mean value and variance of the distribution for each measure and currency in two distinct time periods: before and during the pandemic. Our findings reveal distinct shifts in efficiency before and during the COVID-19 pandemic. Specifically, there was a clear increase in the weak-form inefficiency of traditional currencies during the pandemic. Among cryptocurrencies, BTC stands out for its behavior, which resembles that of traditional currencies. Moreover, our results underscore the significant impact of COVID-19 on weak-form market efficiency during both upward and downward market movements. These findings could be useful for investors, portfolio managers, and policy makers.

## 1. Introduction

It is well known that sudden “large” shocks cause structural changes in financial markets, which potentially have asymmetric effects on market efficiency, portfolio allocation, and volatile spillovers. A recent example of a sudden and large shock to the markets was the outbreak of the COVID-19 pandemic. On 11 March 2020, the World Health Organization (WHO) declared the outbreak of COVID-19 a global pandemic. The WHO announcement caused panic in financial markets worldwide, resulting in many investors suffering significant losses in a very short period of time. On 12 March 2020, the Nikkei-225, S&P500, and FTSE-100 all plunged by about 4.41%, 9.51%, and 10.87%. During the same period, the Australian dollar (AUD) hit a 17-year low of $0.59215, and the New Zealand dollar reached an 11-year low of $0.5850 cents, while gold fell by about 3.53%. The impact of the announcement even affected more contemporary asset classes such as cryptocurrencies. On 12 March 2020, most of the major cryptocurrencies recorded their lowest prices throughout the pandemic crisis. The outbreak of the COVID-19 pandemic and its impact on financial markets have underscored the importance of discussing market efficiency and whether it varies over time, particularly during periods of uncertainty.

The efficient market hypothesis (EMH) constitutes the cornerstone of financial economics, originating from the pioneering work of Bachelier [1] in the early 20th century and further developed by Eugene Fama in 1970 [2]. In his seminal article, Fama not only defined efficient markets but also introduced a distinction between its three forms: weak, semi-strong, and strong efficiency. The weak form of EMH posits that the current prices of financial assets already incorporate all available historical financial information at any given moment. Consequently, the theory suggests that investors cannot achieve abnormal profits by investing in these assets. This form of EMH implies that prices follow a random walk process. In contrast, the semi-strong form of EMH extends the weak form by asserting that financial asset prices not only reflect all historical information (as in the weak form) but also rapidly and unbiasedly incorporate any new public information released in the market. The strong form of EMH takes this concept further, asserting that prices encompass all available information, including historical financial data (as in the weak form), all new public information (as in the semi-strong form), and all private information concerning a financial asset. Numerous studies have sought to test these three forms of EMH, and the results have predominantly refuted the semi-strong and strong forms, as they are not consistently supported by financial data. However, opinions remain divided regarding the weak form of EMH, which encompasses the random walk theory [3]. Furthermore, alternative approaches have emerged to address this issue. One such approach is the adaptive market hypothesis (AMH), introduced by Lo in 2004 [4]. The AMH posits that market efficiency is influenced by the reactions of market participants to evolving market conditions. This perspective acknowledges the dynamic nature of efficiency over time, as investors adapt their investment decisions in response to shifting business environments. Another alternative approach is the fractal markets hypothesis (FMH), which was proposed by Peters in 1994 [5]. At its core, it is based on a notion entirely overlooked in EMH—liquidity. According to FMH, liquidity plays a crucial role in providing a smooth pricing process in the market, ensuring stability. If liquidity diminishes, the market becomes unstable, leading to extreme movements [6,7].

The issue of market efficiency has been explored in the stock market, foreign exchange market (Forex Market, FX Market), futures market, cryptocurrency market, and other markets. From the perspective of measuring efficiency, the existing literature can be divided into two parts: the methods of econometrics and econophysics. In the area of econometrics, there are several popular methods of testing market efficiency, e.g., the Ljung–Box test [8], the runs test [9], the Bartel’s test [10], the wild-bootstrapped automatic variance ratio test [11], the spectral shape tests [12,13], the BDS test [14], the robustified portmanteau test [15], the generalized spectral test [16], fractional unit roots tests [17], etc. On the other hand, in the area of econophysics, many useful and meaningful approaches to test market efficiency have also been developed. A brief description of such approaches can be described as follows. The rescaled-range (R/S) analysis has been used to investigate the long-term memory of returns [18,19]. Various measures of entropy, such as fuzzy, approximate, Tsallis, and Shannon entropy, have been used to investigate the degree of disorder, randomness, and complexity of returns [20,21,22]. Detrended fluctuation analysis (DFA), multifractal detrended fluctuation analysis (MF-DFA), and asymmetric multifractal detrended fluctuation analysis (A-MF-DFA) have been applied to detect the long-range correlations of returns. An intriguing topic that captivates the scientific community involves employing methods like those mentioned earlier to assess market efficiency during crises. In this study, our focus centers on understanding the pandemic’s impact on market efficiency from the viewpoint of econophysics.

The study of the effects of the recent COVID-19 crisis on market efficiency has attracted the interest of many researchers. For example, Wang J. and Wang X. [23] estimated the market efficiency of the S&P 500 Index, gold, Bitcoin, and US dollar index during the extreme event of the COVID-19 pandemic using a multiscale entropy-based method for the scales of hourly and 1 to 30 business days. Their results indicated that at all scales, the four markets’ efficiency decreased sharply and persistently during February and March 2020. Also, market efficiency decreased the most in the S&P 500 Index and the least in the Bitcoin market. Hence, they concluded that Bitcoin market efficiency was more resilient than others during the extreme event, which is an attractive feature to serve as a safe haven asset. Frezza et al. [24], using the multifractional Brownian motion as a model of the price dynamics, analyzed the impact of the COVID-19 pandemic on the efficiency of fifteen financial markets in Europe, the US, and Asia. They found that Asian markets (Hang Seng, Nikkei 225, Kospi) recovered full efficiency, while European and US markets had not yet returned to their precrisis levels of efficiency. Mnif et al. [25] applied the MF-DFA approach to study the level of cryptocurrency efficiency before and after the coronavirus pandemic using a limited time period, until 19th May 2020. Their empirical results indicated that COVID-19 has a positive impact on the market efficiency of the five cryptocurrencies they studied. A similar approach was followed by Aslam et al. [26], who applied MF-DFA to study the efficiency of forex markets during the initial period of the pandemic. In their analysis, they used high-frequency data of six major currencies during the period of 1 October 2019 to 31 March 2020. Before applying MF-DFA, the authors examined the inner dynamics of multifractality through seasonal and trend decomposition using loess. Their results indicated that the efficiency of forex markets during the COVID-19 outbreak declined. Naeem et al. [27] investigated the asymmetric efficiency of four cryptocurrencies (Bitcoin, Ethereum, Litecoin, and Ripple) during the period of 1 July 2017 to 1 April 2020 using hourly data. For their analysis, they utilized A-MF-DFA. Their results showed significant asymmetric multifractality in the price of cryptocurrencies, as well as that the upward trends exhibited stronger multifractality than downward trends. In addition, they applied the time-varying market deficiency measure. These results showed that the levels of inefficiency during the COVID-19 period increased significantly for all four cryptocurrencies. It was, therefore, concluded that the COVID-19 epidemic adversely affected the efficiency of the four cryptocurrencies. Kakinaka and Umeno [28], applying A-MF-DFA, examined the asymmetric multifractality and market efficiency of two cryptocurrencies (Bitcoin, Ethereum) during the COVID-19 pandemic, accounting for different investment horizons. Their results showed that the outbreak affected the efficiency property of price behaviors differently between short- and long-term horizons. More specifically, after the outbreak, the markets exhibited stronger multifractality in the short term but weaker multifractality in the long term. In addition, they studied the asymmetric market patterns between small and large price fluctuations and between upward and downward trends. These results confirmed that the outbreak greatly changed the level of asymmetry in cryptocurrency markets. Mensi et al. [29] examined the impact of the COVID-19 pandemic crisis on the pricing efficiency and asymmetric multifractality of major asset classes (S&P 500, US Treasury bonds, the US dollar index, Bitcoin, Brent oil, and gold) within a dynamic framework. By applying the permutation entropy to intraday data spanning from 30 April 2019 to 13 May 2020, they demonstrated that the pandemic exacerbated the inefficiency of all markets except for Bitcoin. Drożdż et al. [30] investigated the complexity of the cryptocurrency market around the COVID-19 outbreak. In their study, they employed three distinct perspectives. Specifically, they utilized the following: first, the multifractal formalism; second, cross-correlation analyses based on fractal analysis; and third, the topology of a minimal spanning tree of the market. Their results, among other findings, revealed that throughout the considered interval, exchange rate returns exhibited multifractality with intermittent signatures of bifractality. These bifractal characteristics were associated with the most volatile periods of market dynamics, such as the onset of a bull market in April 2019 and the COVID-19 outbreak in March 2020. G. Espinosa-Paredes et al. [31] explored the impact of the COVID-19 outbreak on the efficiency of the crude oil market. They utilized the singular value decomposition (SVD) entropy method in their analysis. The results of their investigation indicate a growing potential for predicting crude oil prices and achieving abnormal returns during the COVID-19 pandemic. Almeida et al. [32] evaluated integration and contagion in the cryptocurrency market in the context of the COVID-19 pandemic, employing two entropy-based measures: mutual information and transfer entropy. Both methodologies revealed mixed levels of integration for cryptocurrencies before and after the onset of the pandemic. Furthermore, their analyses indicated no contagion from the pandemic turmoil to these financial assets. Thus, they concluded that cryptocurrencies may be good investment options during real global shocks, such as the one triggered by the COVID-19 outbreak. Lahmiri and Bekiros [21] examined the changes in informational efficiency across 45 cryptocurrency markets and 16 international stock markets before and during the COVID-19 pandemic. They utilized two robust measures, the largest Lyapunov exponent (LLE) based on Rosenstein’s method and approximate entropy (ApEn), to analyze the price time series. These measures were employed to assess the levels of stability and irregularity in the cryptocurrency and international stock markets. Their findings revealed, among other observations, that cryptocurrencies exhibited higher levels of instability and irregularity during the COVID-19 pandemic compared with the international stock markets. Although the impact of the pandemic on market efficiency is a well-studied topic in the literature, there are still issues that are relatively unexplored.

A characteristic feature of financial markets is heterogeneity because they consist of diverse agents who react differently to incoming information. For instance, information considered negative for one agent, signaling a selling opportunity, might be viewed as a buying opportunity for another agent, and vice versa. However, in many cases, agents exhibit homogeneous behavior, resembling a herd [33]. Understanding the general behavior of market participants is particularly crucial, as phenomena such as herd behavior often contribute to the emergence of inefficient markets [34]. Quantifying the level of randomness in financial markets provides insight into the overall behavior of participants. Randomness is typically characterized by the absence of discernible patterns. A financial market is considered somewhat predictable if it consistently follows the same price patterns and entirely random if there is no repetition of patterns, with participants buying or selling without any discernible pattern [35]. In this context, entropy serves as a statistical measure of the randomness level of a time series based on the quantification of the existence of patterns and their repetitions. Low levels of this statistic measure indicate the existence of such patterns, while high values suggest randomness and unpredictability. Another measure used to estimate the level of complexity or randomness in a time series is Fisher information. This measure behaves inversely to entropy; as complexity or randomness increases, entropy increases, while Fisher information decreases. Additionally, useful conclusions about the randomness of a time series can be obtained by studying its degree of multifractality. Specifically, the more random a time series is, the more unifractal its scaling, indicating that a more multifractal time series is farther from “randomness” [20,36]. Consequently, the methods outlined above are suitable for testing the efficient market hypothesis (EMH) in its weak form, as they can provide valuable information about the randomness of asset returns.

In this article, we explore the impact of the COVID-19 pandemic on the weak-form efficiency of cryptocurrencies and foreign exchange markets. To study weak-form efficiency, we applied a combined approach, for the first time to our knowledge, using the asymmetric MDM method based on A-MF-DFA, fuzzy entropy, Tsallis entropy, and Fisher information. We selected these four measures because, although they are based on different fundamental principles, they all provide information about the degree of randomness or disorder in a financial time series. The motivation for our study is twofold. The first is to determine whether the efficiency of some major currencies in two markets that play a crucial role in the modern financial system was affected by the pandemic. Thus, we initially estimated the temporal evolution of the four mentioned measures using overlapping sliding windows and then examined the mean value of the distribution of each measure in two time periods, before and during the pandemic. We applied this process for each of the measures we used and for each currency separately. By following this approach, which as far as we know is the first time it has been applied in the analysis of market efficiency, we were able to conduct an in-depth comparative analysis among all the currencies we studied and revealed interesting conclusions about how each currency responded individually to the pandemic. Additionally, by studying the mean value of the distributions of the four measures before and during the pandemic, we obtained valuable insights into whether efficiency changed over time, adapting to an uncertain financial environment, as was the case during the COVID-19 period. Secondly, we aim to investigate whether the pandemic caused significant changes in the efficiency levels of each currency. Thus, in addition to the mean value, we also examined the variance of the distributions of each measure for the two periods: before and during the pandemic. In this way, we consider that we are providing a comprehensive picture of the pandemic’s impact on the efficiency of the currencies we studied. In summary, the goal of our study is, through comparative analysis between cryptocurrencies and foreign exchange markets, to fill a gap in the literature, as there has been no in-depth comparative analysis between these two markets to date. An interesting exception is found in the article by Drożdż et al. [37], in which they investigated multiscale cross-correlations involving Bitcoin (BTC), Ethereum (ETH), the euro (EUR), and the US dollar (USD) between 1 July 2016 and 31 December 2018. Additionally, it aims to provide valuable findings for investors, researchers, and regulatory authorities, helping them improve their understanding of the behavior of these two markets before and during periods of uncertainty, such as the COVID-19 period.

## 2. Methods

In this section, we briefly present the A-MF-DFA method (Section 2.1) and the market deficiency measure (MDM) that estimates the degree of market efficiency (Section 2.2). We also briefly introduce fuzzy entropy (Section 2.3), Tsallis entropy (Section 2.4), and Fisher information (Section 2.5), which estimate the degree of randomness of the return time series.

### 2.1. Asymmetric Multifractal Detrended Fluctuation Analysis (A-MF-DFA)

The A-MF-DFA method extends the MF-DFA method by considering positive and negative market trends [38,39]. First, the profile time series of each return time series ${\{x}_{j\;}:j=1,\ldots ,N\}$ are calculated as $X\left(t\right)=\sum_{j=1}^{t}\left(x_{j}-\bar{x}\right)$ for t=1,…, N, where $\bar{x}$ is the average over the entire return time series. Then, the profile time series and the return time series are divided into $N_{n}=\lfloor N/n\rfloor$ non-overlapping segments of length n. In the case that N is not a multiple of n, the division is repeated starting from the other end of the time series to take into account all the available data, making a total of $2N_{n}$ segments for both the profile and the return time series.

Next, for each segment $v=1,\ldots ,\;2N_{n}$, the local trend of the profile series ${\tilde{X}}_{v}\left(i\right),\;i=1,\ldots ,n$ is calculated by fitting a least-square degree-2 polynomial in order to detrend the corresponding profile $X_{v}\left(i\right),\;i=1,\ldots ,n.$ For the return time series, we calculate the local linear trend for each segment to determine whether the return time series shows an uptrend or downtrend. The different trends depend on the sign of the slope $b_{n,v}\neq 0$, where $b_{n,v}$ represents the coefficient of the linear trend for segment v at scale n [28]. If $b_{n,v}>0$ ($b_{n,v}<0$), the return time series have an upward (downward) trend within the vth segment.

Then, we define the residual variance as follows:

(1) $$F^{2}\left(n,\;v\right)=\frac{1}{n}\sum_{i=1}^{n}{\left(X_{v}\left(i\right)-{\tilde{X}}_{v}(i)\right)}^{2}.$$

The asymmetric qth order average fluctuation functions are then calculated by taking the average over the corresponding segments:

(2) $$F_{q}^{+}\left(n\right)={\left\{\frac{1}{M^{+}}\;\sum_{v=1}^{2N_{n}}\frac{1+\mathrm{sgn}(b_{n,\;v})}{2}{\left[F^{2}\left(n,\;v\right)\right]}^{\frac{q}{2}}\right\}}^{\frac{1}{q}},$$

(3) $$F_{q}^{-}\left(n\right)={\left\{\frac{1}{M^{-}}\;\sum_{v=1}^{2N_{n}}\frac{1-\mathrm{sgn}(b_{n,\;v})}{2}{\left[F^{2}\left(n,\;v\right)\right]}^{\frac{q}{2}}\right\}}^{\frac{1}{q}},$$

where $M^{+}=\sum_{v=1}^{2N_{n}}\left(1+\mathrm{sgn}(b_{n,v})\right)/2$ and $M^{-}=\sum_{v=1}^{2N_{n}}\left(1-\mathrm{sgn}(b_{n,v})\right)/2$ denote the number of total segments with directional trends, respectively. Note that for all, $v=1,\ldots ,2N_{n}$, $M^{+}+M^{-}=2N_{n}$ holds. Therefore, the qth order average fluctuation functions for the overall trend is written as:

(4) $$F_{q}\left(n\right)={\left\{\frac{1}{2N_{n}}\sum_{v=1}^{2N_{n}}{\left[F^{2}\left(n,\;v\right)\right]}^{\frac{q}{2}}\right\}}^{1/q}.$$

The calculation is repeated to find the fluctuation function for all box sizes n. If long-range power-law correlations are present, the function will increase with n as a power-law $F_{q}\left(n\right)∼n^{h\left(q\right)},$ where the scaling exponent h(q), namely the generalized Hurst exponent, is calculated as the slope of the linear regression of $\log\left(F_{q}(n)\right)$ versus log⁡(n). The asymmetric generalized exponents $h^{+}(q)$ and $h^{-}\left(q\right)$ are calculated in a similar way using the relationship $F_{q}^{+}\left(n\right)∼n^{h^{+}\left(q\right)}$ and $F_{q}^{-}\left(n\right)∼n^{h^{-}\left(q\right)}.$ In this study, we consider n ranging from 8 to N/4 for the log–log linear regression to estimate the asymmetric generalized Hurst exponents. These exponents are then utilized in the next step to quantitatively calculate the degree of market efficiency.

### 2.2. Market Deficiency Measure (MDM)

If an asset market is efficient, all kinds of fluctuations, including large fluctuations (q=+5) and small fluctuations (q=−5) must follow the random walk process. In other words, h(q)s related to different qs must be equal to 0.5. To measure and compare market efficiency, Wang et al. [40] suggested a measure that illustrates the discrepancy from an efficient market by evaluating the deviation from a random walk process in terms of both large (q=+5) and small (q=−5) fluctuations. In other words, the measure is evaluated in terms of the multifractal degree of the time series. This measure is called “market deficiency measure” and can be estimated as follows [25,39,41,42,43]:

(5) $$MDM=\frac{1}{2}\left(\left|h\left(-5\right)-0.5\right|+\left|h\left(+5\right)-0.5\right|\right).$$

Hence, for an efficient market, the value of the MDM must be equal to zero. On the other hand, if the market is inefficient, the value of the MDM must deviate above zero. Specifically, the higher the MDM value, the higher the multifractality and, thus, the more inefficient the market will be.

In the same vein, asymmetric MDM, $MDM^{\pm }$ can be introduced [28], and we define the measure as follows:

(6) $$MDM^{\pm }=\frac{1}{2}\left(\left|h^{\pm }\left(-5\right)-0.5\right|+\left|h^{\pm }\left(+5\right)-0.5\right|\right).$$

This measure allows us to investigate the degree of efficiency under different market trends.

### 2.3. Fuzzy Entropy

Expanding upon the concepts already established with approximate entropy (ApEn) and sample entropy (SampEn), Chen et al. [44,45] combined elements of fuzzy sets and information theory to develop a fuzzy version of the SampEn. Fuzzy entropy (FuzzyEn), like its ancestors, ApEn and SampEn [45], is a “regularity statistic” that quantifies the (un)predictability of fluctuations in a time series. For the estimation of FuzzyEn, the similarity between vectors is defined based on fuzzy membership functions and the vectors’ shapes. The gradual and continuous boundaries of the fuzzy membership functions lead to a series of advantages, such as the continuity and validity of FuzzyEn at small values, higher accuracy, stronger relative consistency, and even less dependence on the data length. FuzzyEn can be considered as an upgraded alternative to SampEn (and ApEn) for the evaluation of complexity, especially for short time series contaminated by noise [46].

Similar to SampEn, FuzzyEn excludes self-matches. Nevertheless, it applies a slightly different definition for the employed first N−m vectors of a length of m, by removing a baseline ${\bar{s}}_{i}$:

(7) $${\bar{s}}_{i}=m^{-1}\sum_{j=0}^{m-1}s_{i+j},$$

i.e., for the FuzzyEn estimations, we use the first N−m of the vectors:

(8) $$X_{i}^{m}=\left\{s_{i},\;s_{i+1},\ldots ,s_{i+m-1}\right\}-{\bar{s}}_{i},\;\;i=1,\;2,\ldots ,\;N-m+1,$$

Then, the similarity degree, $D_{ij}^{m}$, between each pair of vectors, $X_{j}^{m}$ and $X_{i}^{m}$, being within a distance, r, from each other is defined using a fuzzy membership function:

(9) $$D_{ij}^{m}=\mu \left(d_{ij}^{m},r\right),$$

where $d_{ij}^{m}$ is, as in the case of ApEn and SampEn, the supremum norm difference between $X_{i}^{m}$ and $X_{j}^{m}$. For each vector, $X_{i}^{m}$, we estimate the average similarity degrees with respect to all other vectors, $X_{j}^{m},\;j=1,\;2,\ldots ,N-m+1$, and j≠i (i.e., excluding itself):

(10) $$\phi_{i}^{m}\left(r\right)={\left(N-m-1\right)}^{-1}\sum_{i=1,j\neq i}^{N-m}D_{ij}^{m}.$$

Then, we evaluate:

(11) $$\varphi^{m}\left(r\right)={\left(N-m\right)}^{-1}\sum_{i=1}^{N-m}\phi_{i}^{m}\left(r\right),$$

and

(12) $$\varphi^{m+1}\left(r\right)={\left(N-m\right)}^{-1}\sum_{i=1}^{N-m}\phi_{i}^{m+1}\left(r\right).$$

The FuzzyEn(m,r) is then defined as:

(13) $$FuzzyEn\left(m,r\right)=\underset{N\to \infty }{\lim}\left[\ln\varphi^{m}\left(r\right)-\ln\varphi^{m+1}\left(r\right)\right],$$

which, for finite time series, can be calculated using the statistic:

(14) $$FuzzyEn\left(m,r,N\right)=\ln\varphi^{m}\left(r\right)-\ln\varphi^{m+1}\left(r\right).$$

As mentioned above, FuzzyEn is a measure of the estimation of complexity. More specifically, lower FuzzyEn values demonstrate a greater chance that a set of data will be followed by similar data (regularity). Hence, lower values demonstrate greater regularity. Conversely, a greater value of FuzzyEn indicates a smaller chance of similar data being repeated (irregularity). Thus, greater values convey more randomness, disorder, and system complexity. Consequently, a low (high) value of FuzzyEn reflects a high (low) degree of regularity [47].

### 2.4. Tsallis Entropy

In a vast variety of systems that exhibit long-range interactions or long-term memory or are multifractal in nature, they have been found to be better described by a generalized statistical-mechanical formalism proposed by Tsallis [48,49]. Tsallis, inspired by multifractal concepts, introduced an entropic expression characterized by an index, $q_{TS}$, which leads to non-extensive statistics [48,49]:

(15) $$S_{q_{TS}}=k\frac{1}{q_{TS}-1}\left(1-\sum_{i=1}^{W}p_{i}^{q_{TS}}\right),$$

where $q_{TS}$ is a real number, k is the Boltzmann’s constant from statistical thermodynamics, $p_{i}$ are probabilities associated with the micro-configurations, and W is their total number. It is important to note that there is a remarkable conceptual similarity between Tsallis’ entropy definition and the notion of Rényi entropies.

The entropic index, $q_{TS}$, describes the deviation of Tsallis entropy from the standard Boltzmann–Gibbs entropy. Indeed, using $p_{i}^{\left(q_{TS}-1\right)}=e^{(q_{TS}-1)\ln\left(p_{i}\right)}\;\sim \;1+(q_{TS}-1)\ln\left(p_{i}\right)$ in the limit $q_{TS}\to 1$, we recover the Boltzmann–Gibbs entropy:

(16) $$S_{1}=-k\sum_{i=1}^{W}p_{i}\ln\left(p_{i}\right),$$

as the thermodynamic analog of the information-theoretic Shannon entropy.

For $q_{TS}\neq 1$, the entropic index, $q_{TS}$, characterizes the degree of non-extensivity reflected in the following pseudo-additivity rule:

(17) $$\frac{S_{q_{TS}}(A+B)}{k}=\frac{S_{q_{TS}}(A)}{k}+\frac{S_{q_{TS}}(B)}{k}+(q_{TS}-1)\frac{S_{q_{TS}}(A)}{k}\frac{S_{q_{TS}}(B)}{k},$$

where A and B are two subsystems. In case these subsystems have special probability correlations, extensivity does not hold for $q_{TS}=1\;(S_{1}\neq S_{1}\left(A\right)+S_{1}\left(B\right))$, but may occur for $S_{q_{TS}}$, with a particular value of the index, $q_{TS}\neq 1$. Such systems are called non-extensive [48]. The cases $q_{TS}>1$ and $q_{TS}<1$ correspond to sub-additivity and super-additivity, respectively. As in the case of Rényi entropies, we may think of $q_{TS}$ as a bias parameter: $q_{TS}<1$ privileges rare events, while $q_{TS}>1$ highlights prominent events [50].

It is noted that the parameter, $q_{TS}$, itself is not a measure of the complexity of the system but measures the degree of the non-extensivity of the system. In turn, the temporal variations of the Tsallis entropy, $S_{q_{TS}}$, for some $q_{TS}$ quantify the dynamical changes in the complexity of the system. In particular, lower $S_{q_{TS}}$ values characterize portions of the signal with lower complexity [46]. In this article, for the calculation of Tsallis entropy, we have chosen to use the value $q_{TS}=1.8$ for the non-extensive parameter, $q_{TS}$, after [20,51].

### 2.5. Fisher Information Measure

In the last decades, Fisher information has been increasingly gaining the interest of scientists in different scientific fields. It was first introduced by Fisher [52] as a representation of the amount of information in the results of experimental measurements of an unknown parameter of a stochastic system, or simply the amount of information that can be extracted from a set of measurements (or the “quality” of the measurements) [53]. Fisher information is a useful method for studying non-stationary and complex time series [54]. It is used as a measure of the level of disorder of a system, which behaves inversely to entropy, i.e., when the disorder increases, the entropy increases while the Fisher information decreases. Fisher information has been successfully applied to many different systems, revealing its ability to describe their complexity [54,55,56,57]. Additionally, its use has been suggested to identify reliable precursors of critical events [58,59,60]. Moreover, Fisher information presents the so-called “locality” property in contrast to the “globality” of entropy, which refers to the sensitivity of Fisher information in changes in the shape of the probability distribution corresponding to the measured variable not presented by entropy [61,62]. The Fisher information measure can be expressed as:

(18) $$I_{x}=\sum_{n=1}^{N-1}\frac{{\left[p\left(x_{n+1}\right)-p(x_{n})\right]}^{2}}{p(x_{n})}.$$

The discrete probability distribution $p(x_{n})$ corresponds to the specific values of the unknown underlying probability density function at the center values of the intervals $\left\{x_{n}\right\}$, which are not necessarily of equal length. The probability density function is usually approximated using a histogram or the kernel density estimator technique, which employs different kernel functions such as the Gaussian kernel or Epanechnikov kernel [53].

## 3. Data

The cryptocurrency market is a relatively new and emerging market with a unique trading mechanism that makes it very different from traditional markets [33]. More than 21,800 different cryptocurrencies are currently traded around the world with an estimated total market capitalization of over 843 billion dollars (see, e.g., https://coinmarketcap.com/ (accessed on 12 December 2022)). On the other hand, the foreign exchange market is the largest financial market worldwide, with transactions amounting to trillions of US dollars daily [63,64].

**Figure 1:**
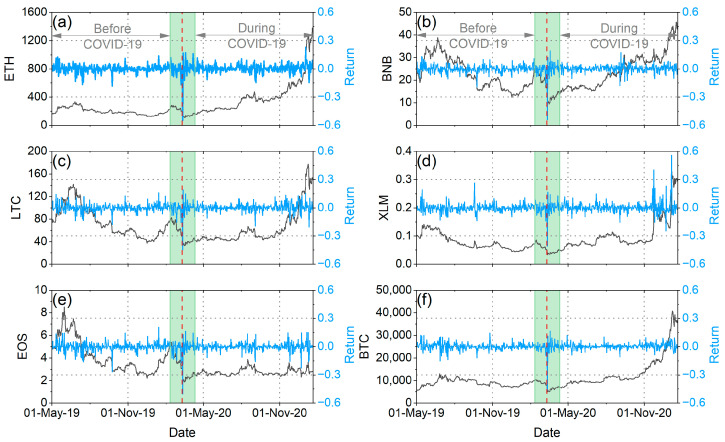
(**a**): Evolution of ETH prices (black curves, left vertical axis) and returns (blue curves, right vertical axis) over the period 1 May 2019 to 20 January 2021. (**b**): Evolution of BNB prices (black curves, left vertical axis) and returns (blue curves, right vertical axis) over the period 1 May 2019 to 20 January 2021. (**c**): Evolution of LTC prices (black curves, left vertical axis) and returns (blue curves, right vertical axis) over the period 1 May 2019 to 20 January 2021. (**d**): Evolution of XLM prices (black curves, left vertical axis) and returns (blue curves, right vertical axis) over the period 1 May 2019 to 20 January 2021. (**e**): Evolution of EOS prices (black curves, left vertical axis) and returns (blue curves, right vertical axis) over the period 1 May 2019 to 20 January 2021. (**f**): Evolution of BTC prices (black curves, left vertical axis) and returns (blue curves, right vertical axis) over the period 1 May 2019 to 20 January 2021. The red vertical dash line corresponds to the date of the WHO announcement (i.e., 11 March 2020). The green shaded area corresponds to the time period excluded from our analyses (i.e., one month before and one month after the WHO announcement).

**Figure 2:**
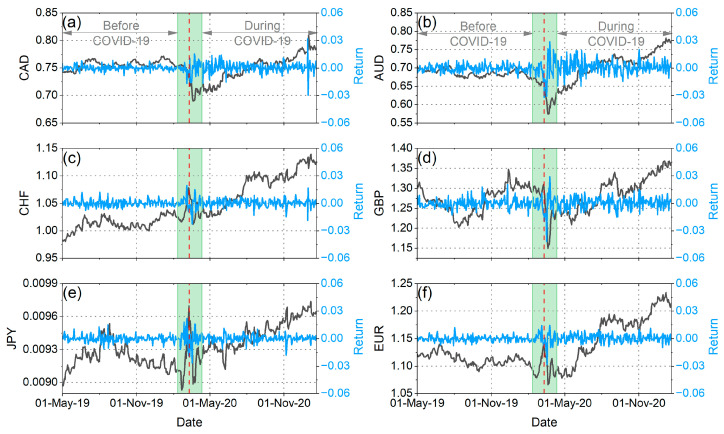
(**a**): Evolution of CAD prices (black curves, left vertical axis) and returns (blue curves, right vertical axis) over the period 1 May 2019 to 20 January 2021. (**b**): Evolution of AUD prices (black curves, left vertical axis) and returns (blue curves, right vertical axis) over the period 1 May 2019 to 20 January 2021. (**c**): Evolution of CHF prices (black curves, left vertical axis) and returns (blue curves, right vertical axis) over the period 1 May 2019 to 20 January 2021. (**d**): Evolution of GBP prices (black curves, left vertical axis) and returns (blue curves, right vertical axis) over the period 1 May 2019 to 20 January 2021. (**e**): Evolution of JPY prices (black curves, left vertical axis) and returns (blue curves, right vertical axis) over the period 1 May 2019 to 20 January 2021. (**f**): Evolution of EUR prices (black curves, left vertical axis) and returns (blue curves, right vertical axis) over the period 1 May 2019 to 20 January 2021. The red vertical dash line corresponds to the date of the WHO announcement (i.e., 11 March 2020). The green shaded area corresponds to the time period excluded from our analyses (i.e., one month before and one month after the WHO announcement).

In our analysis, we applied all the methods presented in Section 2 to the daily logarithmic returns ($r_{t}={lnp}_{t}-{lnp}_{t-1}$, where $p_{t}$ denotes the price at time t) of six cryptocurrencies (Ethereum (ETH), Litecoin (LTC), EOS (EOS), Binance (BNB), Stellar Lumen (XLM), and Bitcoin (BTC)) and six traditional currencies (Canadian dollar (CAD), Swiss franc (CHF), Japanese yen (JPY), Australian dollar (AUD), British pound (GBP), and euro (EUR)) against the US dollar (USD) during the period from 1 May 2019 to 20 January 2021. All financial time series were taken from Yahoo Finance (http://finance.yahoo.com/ (accessed on 1 October 2023)). We split the whole samples into two sub-periods, before and after the pandemic outbreak. In order to study the actual impact of the pandemic on the markets and not the initial panic reactions caused by the shock of the WHO announcement, we decided to remove from our analysis a period of time before and after the announcement (one month before and one month after). Therefore, we considered the period from 1 May 2019 to 11 February 2020 as the period before COVID-19 and the period from 11 April 2020 to 20 January 2021 as the period during COVID-19 (Figure 1 and Figure 2). We have chosen to have the period during COVID-19 stop at the beginning of 2021 because the subsequent time interval was marked by events that significantly influenced the volatility of the cryptocurrency market, events that are not directly related to the pandemic.

## 4. Results

In a perfectly weak-form efficient market, future prices are not predictable because their fluctuations are random. Therefore, the more efficient an asset is, the more random and unpredictable its price dynamics will be. Several methods have been proposed to evaluate whether an asset is weak-form efficient or not. All these methods share the common rationale of measuring the extent to which empirical price dynamics deviate from the assumption of complete randomness (martingale hypothesis). In our study, we applied four popular measures that, although based on different fundamental principles, all provide information about the degree of randomness or disorder of a financial time series. More specifically, we applied the MDM method based on A-MF-DFA, fuzzy entropy, Tsallis entropy, and Fisher information. Interpreting the methods we applied, we expect that the more weak-form efficient an asset is (consequently the more random its price dynamics), the smaller the MDM (increasing randomness) and Fisher information (decreasing information) values will be. Conversely, we anticipate the fuzzy and Tsallis entropy values to be larger (increasing randomness or disorder). On the other hand, the more weak-form inefficient an asset is, the larger we expect the MDM and Fisher information values to be and the smaller we expect the fuzzy and Tsallis entropy values to be.

Utilizing the information provided through the aforementioned methods, we conducted a thorough comparative analysis of the pandemic’s impact on the weak-form efficiency of both the cryptocurrency and forex markets. To assess whether the weak-form efficiency levels of the two markets have significantly changed since the onset of the COVID-19 pandemic, as well as whether the pandemic has caused significant changes in weak-form efficiency levels, we employed an approach that, to the best of our knowledge, is being applied for the first time in the estimation of market efficiency. We assessed the time evolution of asymmetric weak-form efficiency using the MDM approach, as well as the time evolution of randomness or disorder using fuzzy entropy, Tsallis entropy, and Fisher information, all with overlapping sliding windows (where the window length was set at 512 samples and the sliding step was 1 sample). Next, we examined the distributions of the time evolution of the aforementioned measures both before and during the COVID-19 period using violin plots. Furthermore, we applied Student’s *t*-test (for means) and an F-test (for variances) to the estimated sample populations of the MDM, fuzzy entropy, Tsallis entropy, and Fisher information measures to achieve more accurate results. These statistical tests were conducted on two levels: firstly, to assess differences between time periods (i.e., before and during the pandemic period), and secondly, to explore disparities between the cryptocurrency and forex markets. All statistical tests were executed at a 5% significance level, i.e., we rejected a null hypothesis when the test *p*-value was less than 5%.

**Figure 3:**
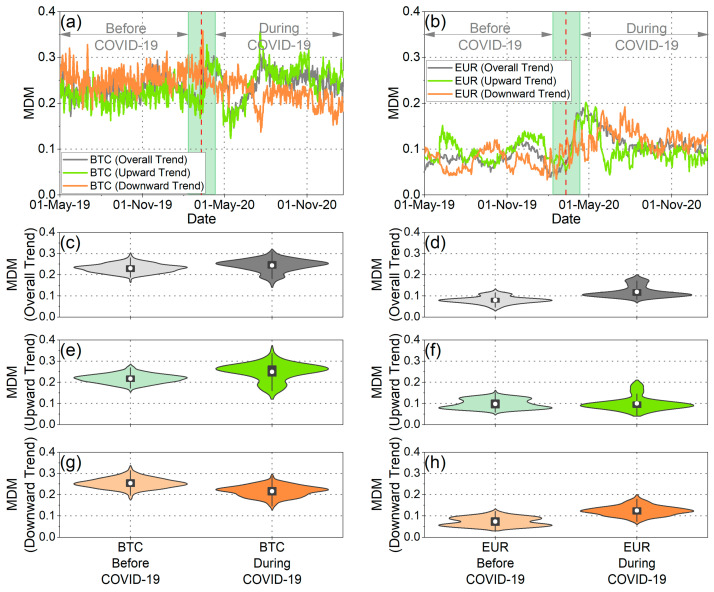
(**a**) Temporal evolution of BTC efficiency in overall, upward, and downward market trends using asymmetric MDM. (**b**) Temporal evolution of EUR efficiency in overall, upward, and downward market trends using asymmetric MDM. (**c**,**d**) The violin plots illustrate the asymmetric MDM values distribution of BTC and EUR in the period before and during the COVID-19 pandemic for the overall market trends. (**e**,**f**) The violin plots illustrate the asymmetric MDM values distribution of BTC and EUR in the period before and during the COVID-19 pandemic for the upward market trends. (**g**,**h**) The violin plots illustrate the asymmetric MDM values distribution of BTC and EUR in the period before and during the COVID-19 pandemic for the downward market trends.

Initially, we concentrated on comparing BTC with EUR, and subsequently, we expanded our analysis to five more cryptocurrencies and five more traditional currencies. Figure 3a,b illustrate the MDM values for BTC and EUR across all market trends, including overall, upward, and downward trends. To visually depict the distribution of MDM values for BTC and EUR before and during the pandemic, we employed violin plots, a visualization technique that combines a box plot with a kernel density plot, as demonstrated in Figure 3c–h.

As observed across overall market trends, the MDM values associated with EUR exhibit a significantly lower mean compared to those of BTC, both before and during the pandemic. These findings were further substantiated through the application of a one-sided *t*-test, the results of which are comprehensively presented in Table 1. The outcomes of the *t*-test, conducted both before and during the COVID-19 pandemic, indicate the rejection of the null hypothesis, which posits that the mean of MDM values for BTC is smaller than that of EUR. Conversely, the reverse null hypothesis is not rejected. Hence, it is deduced from both the violin plots and the statistical tests that the mean of MDM values for EUR is significantly lower compared to that for BTC, and this conclusion holds for both pre-pandemic and pandemic periods. Therefore, it is evident that across overall market trends, BTC was more inefficient than EUR both before and during the pandemic. This notable discrepancy in efficiency levels between BTC and EUR may be attributed to potential disparities in investor confidence in the former relative to the latter. It is worth noting that investors could consider this outcome when making asset allocation decisions and conducting portfolio risk assessments.

When examining each currency individually, it becomes apparent that when analyzing the overall market trend (Figure 3c,d), the means of the MDM values for both BTC and EUR during the pandemic period were higher than the mean values of the previous period. Hence, it appears that the pandemic rendered both BTC and EUR more inefficient. This observation suggests that market efficiency has changed over time, which aligns with Lo’s adaptive market hypothesis [4]. Therefore, we can conclude that investors adapted their investment decisions in response to the changing financial environment created mainly after the outbreak of the pandemic. Furthermore, this escalation in inefficiency within both BTC and EUR during the pandemic period aligns with the conclusions of a recent study [20], in which the authors documented a reduction in complexity and an augmentation in multifractality in both BTC and EUR returns. The heightened inefficiency further substantiates the notion that both returns exhibited reduced randomness following the outbreak of the pandemic.

When we focus on the variance of the distributions, we observe, from both the violin plots and the F-test, that the variance of the MDM values for BTC was greater than that of the MDM values for EUR before the pandemic (Figure 3c,d and Table 2). Consequently, the efficiency of EUR does not fluctuate as strongly as that of BTC. On the other hand, during the pandemic, it appears that the variance of both BTC and EUR distributions increased. This observation implies that the pandemic had a considerable impact on the efficiency levels of each currency.

Furthermore, we conducted an analysis of the time evolution of the MDM, taking into account the asymmetric behavior of market fluctuations, specifically during upward trends (when prices tend to increase) and downward trends (when prices tend to decrease). Applying the asymmetric analysis, it was confirmed that the values vary depending on market trends, resulting in differences in efficiency between rising and falling markets. This finding aligns with the conclusions of Mnif et al. [25], Naeem et al. [27], and Kakinaka and Umeno [28], which suggest that financial time series exhibit notable asymmetric patterns. As depicted in the violin plots in Figure 3e–h, along with the results presented in Table 1, the asymmetric MDM revealed different traces of efficiency before and during the pandemic. Upon examining the mean of the distribution, noteworthy results emerge. Firstly, it is evident that, both before and during the pandemic, for both upward and downward market trends, the mean of the MDM values for BTC surpasses the corresponding mean of the MDM values for EUR. This finding supports the conclusion that EUR exhibited greater efficiency than BTC across all market trends. This assertion is further validated using the one-sided *t*-test, where the null hypothesis, suggesting that the mean of the MDM values for BTC is less than that of EUR, is rejected for both upward and downward market trends (Table 1). Conversely, the reverse null hypothesis is not rejected (Table 1). Another interesting aspect revealed by the study of the mean values of asymmetric MDM distributions for BTC and EUR is as follows: on one hand, the mean of the MDM values for BTC during upward market trends (as well as for the overall market trend) increased during the COVID-19 period. On the other hand, for the downward market trends of BTC, the mean value decreased. In other words, BTC demonstrated increased efficiency during falling markets, while in all the other cases, market inefficiency increased. As for EUR, the mean value of the MDM during downward market trends increased during the pandemic period, indicating that EUR became less efficient in falling markets. Conversely, for upward market trends, the mean value of the MDM remained consistent both before and during the pandemic. These findings indicate that the predictability of future returns for EUR was higher during market declines following the pandemic outbreak compared to the pre-pandemic period. Conversely, the predictability of future returns for BTC was lower during falling markets after the pandemic outbreak in comparison to the pre-pandemic period.

Analyzing both the upward and downward market trends, it is evident that during the pandemic period, the variance of the MDM values for BTC exceeded that of EUR in both market trends (Figure 3e–h and Table 2). Furthermore, a comparison of the variances of the MDM values for BTC and EUR in the downward market trends before COVID-19 reveals a greater variance for BTC. Conversely, in the upward market trends, our analysis leads to the conclusion that the variances of the MDM values for BTC and EUR are statistically indistinguishable, as neither of the two null hypotheses—namely, that the variance of MDM values for BTC is smaller than that of EUR or vice versa—can be rejected. Additionally, upon closer examination of the variance in MDM values for BTC, it becomes apparent that this variance increased during the pandemic in both upward and downward market trends. Conversely, in the case of EUR, the variance in the downward market trends remained consistent before and during COVID-19, while it exhibited an increase in the upward market trends during the pandemic period. These findings collectively underscore the substantial impact of COVID-19 on market efficiency, influencing both rising and falling markets.

**Figure 4:**
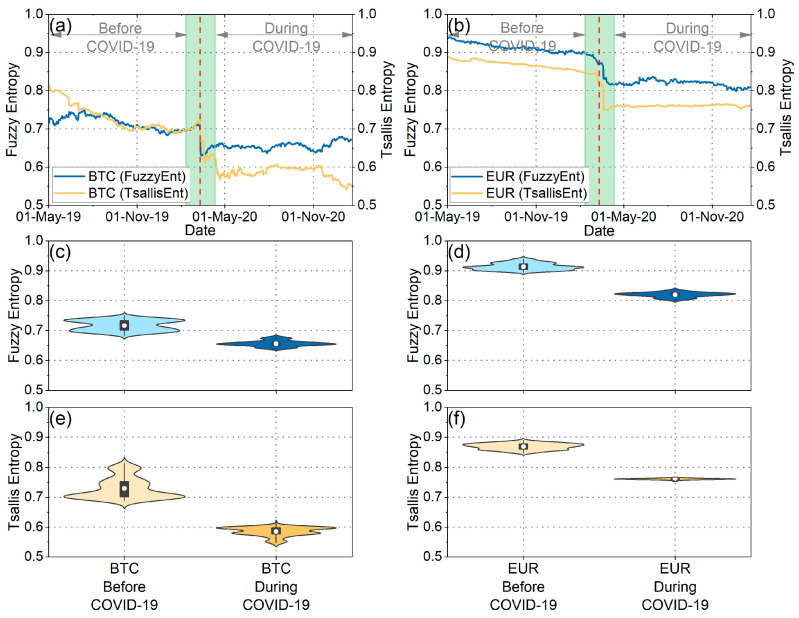
(**a**) Time evolution of fuzzy and Tsallis entropies of BTC returns. (**b**) Time evolution of fuzzy and Tsallis entropies of EUR returns. (**c**,**d**) The violin plots illustrate the distribution of fuzzy entropy values for BTC and EUR returns before and during the COVID-19 pandemic. (**e**,**f**) The violin plots illustrate the distribution of Tsallis entropy values for BTC and EUR returns before and during the COVID-19 pandemic.

**Figure 5:**
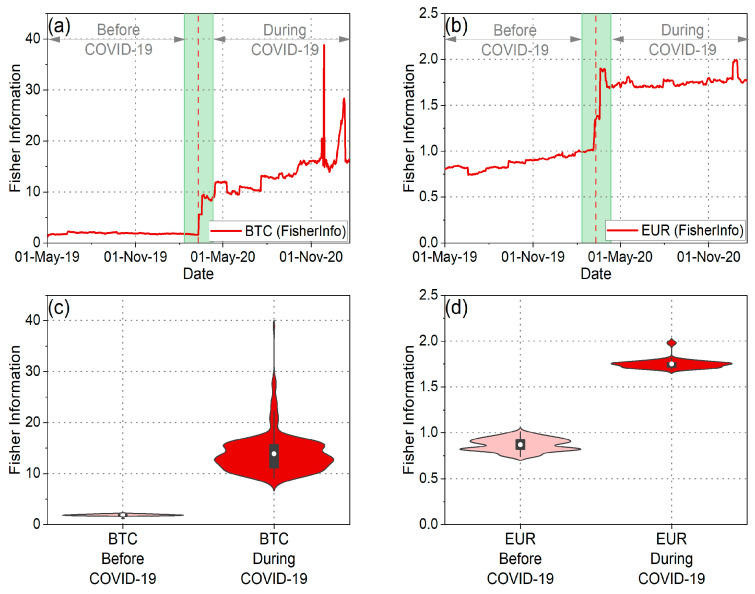
(**a**) Time evolution of Fisher information of BTC returns. (**b**) Time evolution of Fisher information of EUR returns. (**c**,**d**) The violin plots illustrate the distribution of Fisher information values for BTC and EUR returns before and during the COVID-19 pandemic.

Subsequently, our analysis extended to assessing the weak-form efficiency of BTC and EUR through the utilization of fuzzy entropy, Tsallis entropy, and Fisher information. Figure 4a,b depict fuzzy entropy and Tsallis entropy, while Figure 5a,b illustrate Fisher information for both BTC and EUR. Additionally, Figure 4c–f and Figure 5c,d illustrate the distributions of these measures during both the pre-pandemic and pandemic periods.

As observed in the violin plots and the statistical tests (Table 3), the mean values of fuzzy entropy and Tsallis entropy decreased, while the mean value of Fisher information increased during the pandemic period for both BTC and EUR. These findings indicate that all complexity measures suggest a reduction in the randomness and disorder of the time series for BTC and EUR returns during the pandemic period. Consequently, it is inferred that the pandemic rendered BTC and EUR less efficient and, therefore, more predictable. This likely suggests that investors exhibited a more “organized” and similar behavior during this period, reducing the complexity of both markets. Corresponding results have been presented in the article [20]. Additionally, it is noteworthy that throughout the entire analysis period, BTC returns exhibited lower randomness than the corresponding EUR returns. Entropies displayed lower mean values, while Fisher information mean values were significantly higher. These findings lead to the conclusion that BTC returns were more predictable than the corresponding EUR returns both before and during the pandemic.

Furthermore, intriguing insights emerge from the analysis of the distributions’ variance (Figure 4 and Figure 5 and Table 4). Specifically, examining the distribution of Fisher information values reveals that during the pandemic, the randomness of BTC returns showed significantly increased volatility compared to the previous period. This observation signifies the significant impact of the pandemic on the volatility of BTC efficiency, aligning with the findings derived from the efficiency analysis using the MDM method. On the other hand, the variance in Fisher information values for EUR seems to have decreased during the pandemic compared to the previous period. This finding suggests that although efficiency levels changed significantly during the pandemic compared to the previous period, there were no significant changes in the efficiency of the EUR during the pandemic period. Similar results for EUR are observed when studying the fuzzy and Tsallis entropies. Also, when examining the fuzzy and Tsallis entropies in the case of BTC, it becomes evident that its values exhibit smaller fluctuations during the pandemic compared to the preceding period.

**Figure 6:**
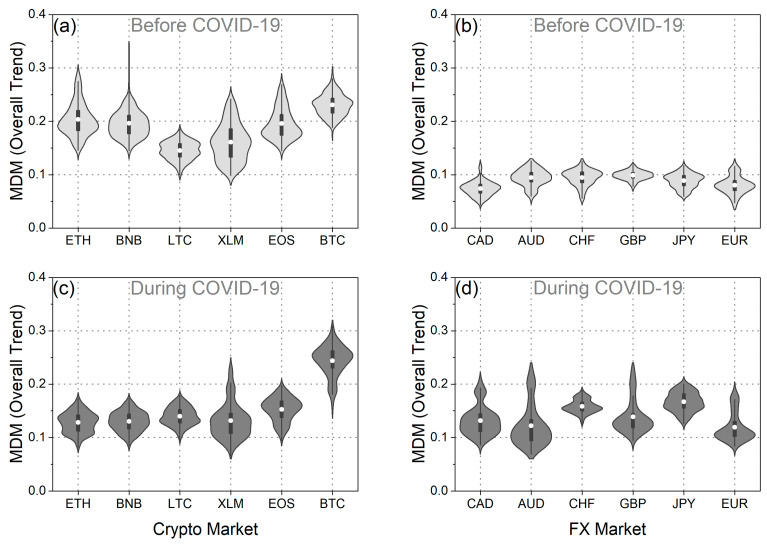
The violin plots of the MDM value distribution for the overall market trend of: (**a**) cryptocurrency in the period before COVID-19, (**b**) traditional currency in the period before COVID-19, (**c**) cryptocurrency during the COVID-19 period, and (**d**) traditional currency during the COVID-19 period.

**Figure A1:**
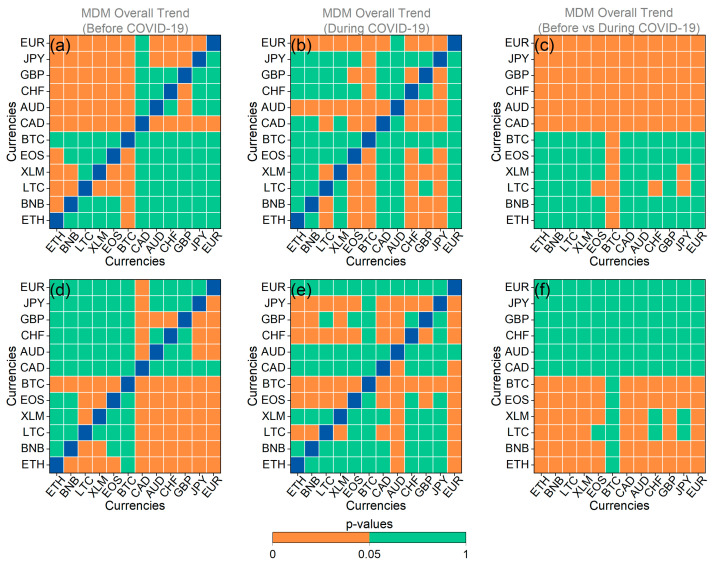
Each heatmap displays one-sided *t*-test probability values. All statistical tests were conducted at a 5% significance level. In the heatmaps: orange color signifies acceptance of the null hypothesis. Green color indicates rejection of the null hypothesis. Blue color represents cases where the statistical test is not applicable due to the comparison of identical currencies. Specifically: (**a**) Null hypothesis: the mean value of MDM before the pandemic for each currency on the Y-axis is greater than the mean value of MDM before the pandemic for each currency on the X-axis. (**b**) Null hypothesis: the mean value of MDM during the pandemic for each currency on the Y-axis is greater than the mean value of MDM during the pandemic for each currency on the X-axis. (**c**) Null hypothesis: the mean value of MDM before the pandemic for each currency on the Y-axis is greater than the mean value of MDM during the pandemic for each currency on the X-axis. (**d**) Reverse null hypothesis: the mean value of MDM before the pandemic for each currency on the Y-axis is less than the mean value of MDM before the pandemic for each currency on the X-axis. (**e**) Reverse null hypothesis: the mean value of MDM during the pandemic for each currency on the Y-axis is less than the mean value of MDM during the pandemic for each currency on the X-axis. (**f**) Reverse null hypothesis: the mean value of MDM before the pandemic for each currency on the Y-axis is less than the mean value of MDM during the pandemic for each currency on the X-axis.

Next, we extended our analysis to five additional cryptocurrencies and five more traditional currencies. It is important to note that from this point on, we chose to present the statistical tests as heatmap plots because we believe that visualizing the results for all currencies together is more straightforward through figures than through tables (see Appendix A). As observed across the overall market trends (Figure 6a,b and Figure A1a,d), in the period preceding the pandemic, cryptocurrencies exhibited significantly higher mean values of MDM than traditional currencies. Consequently, it can be deduced that cryptocurrencies were markedly more inefficient than traditional currencies before the pandemic. The distinct contrast in efficiency levels between the two markets likely arises from the cryptocurrency market’s relatively smaller size compared to the forex market, as well as the fact that the cryptocurrency market is characterized by fewer regulations and financial reforms than the forex market. Also, BTC and CAD are of particular interest, because the first appears to have been the most inefficient among all the coins we studied, while the second was the most efficient. Additionally, it is noteworthy that all the traditional currencies demonstrated very similar mean values of MDM, indicating similar levels of efficiency. Conversely, cryptocurrencies did not exhibit such uniform behavior, suggesting varying degrees of efficiency among them.

During the pandemic period, traditional currencies displayed a higher mean of MDM values compared to the pre-COVID-19 period, as depicted in Figure 6c,d, and corroborated using statistical tests (Figure A1b,e). Thus, it can be inferred that the efficiency of the forex market declined during the COVID-19 outbreak, a finding congruent with the results of Aslam et al. [26], who noted, among other things, a decrease in the efficiency of foreign exchange markets during the pandemic. Furthermore, it appears that the consistent behavior observed among traditional currencies before the pandemic did not persist during the pandemic period, as some currencies exhibited significantly different mean values of MDM compared toothers (e.g., CHF compared to EUR). Additionally, during the same period, all cryptocurrencies, except BTC, displayed a common behavior in terms of efficiency, with lower means than the period before the pandemic, indicative of increased efficiency. These findings are partially in line with the results of Mnif et al. [25], who noted an increase in the efficiency of ETH, LTC, BNB, and BTC during the pandemic compared to the previous period. However, in the case of BTC, our results do not align with theirs. This discrepancy likely arises from the fact that the time period we studied after the pandemic outbreak is considerably longer compared to the period studied by Mnif et al. [25]. On the other hand, our findings regarding the behavior of BTC align with the analysis of Kakinaka and Umeno [28], who observed a decrease in the efficiency of Bitcoin during the pandemic period compared to the previous period, particularly in the short-term horizon.

Finally, when comparing the efficiency of all currencies before and during the pandemic, intriguing insights emerge. In Figure 6 and Figure A1c,f, we observe a clear differentiation between cryptocurrencies and traditional currencies. Specifically, traditional currencies appeared to be more efficient before the pandemic when compared to all currencies during the pandemic. In contrast, the majority of cryptocurrencies became more efficient during the pandemic compared to all other currencies before the pandemic. An interesting exception was BTC; unlike the other cryptocurrencies, where efficiency increased during the pandemic, BTC’s efficiency decreased. This finding is noteworthy because it sets BTC apart from other cryptocurrencies. Additionally, it highlights that the pandemic impacted BTC’s efficiency in a similar manner to traditional currencies, which also became less efficient. Therefore, this result contributes to the ongoing debate on BTC maturity signs [65,66].

**Figure A2:**
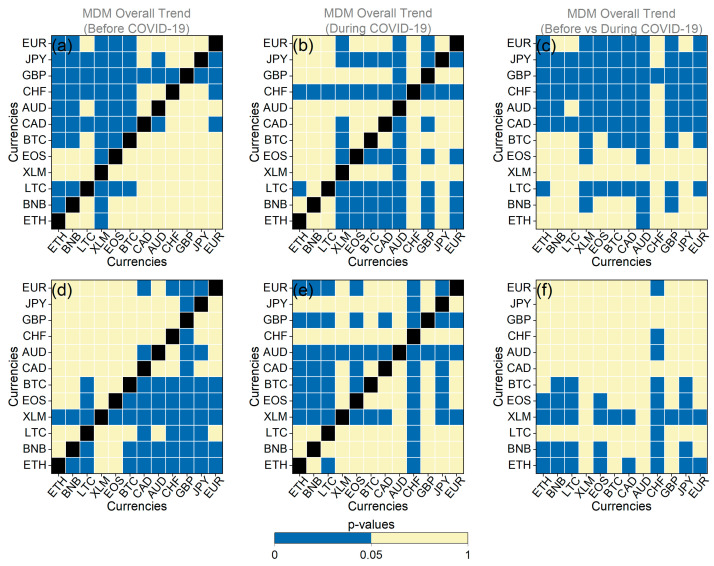
Each heatmap displays F-test probability values. All statistical tests were conducted at a 5% significance level. In the heatmaps: yellow color signifies acceptance of the null hypothesis, blue color indicates rejection of the null hypothesis, and black color represents cases where the statistical test is not applicable due to the comparison of identical currencies. Specifically: (**a**) Null hypothesis: the variance of MDM before the pandemic for each currency on the Y-axis is greater than the variance of MDM before the pandemic for each currency on the X-axis. (**b**) Null hypothesis: the variance of MDM during the pandemic for each currency on the Y-axis is greater than the variance of MDM during the pandemic for each currency on the X-axis. (**c**) Null hypothesis: the variance of MDM before the pandemic for each currency on the Y-axis is greater than the variance of MDM during the pandemic for each currency on the X-axis. (**d**) Reverse null hypothesis: the variance of MDM before the pandemic for each currency on the Y-axis is less than the variance of MDM before the pandemic for each currency on the X-axis. (**e**) Reverse null hypothesis: the variance of MDM during the pandemic for each currency on the Y-axis is less than the variance of MDM during the pandemic for each currency on the X-axis. (**f**) Reverse null hypothesis: the variance of MDM before the pandemic for each currency on the Y-axis is less than the variance of MDM during the pandemic for each currency on the X-axis.

Furthermore, an analysis of the variance within the distributions of MDM values also yields interesting insights. Specifically, it becomes evident that during the pre-pandemic period, traditional currencies maintained a relatively consistent degree of efficiency. In contrast, the variance within the distributions of MDM values for cryptocurrencies was significantly larger, displaying notable volatility. The only exception to this pattern was LTC, which exhibited a divergence almost equivalent to that of AUD and EUR in the pre-pandemic period (Figure A2a,d). Conversely, during the pandemic period, the variance within the distributions of MDM values for most cryptocurrencies, with the exception of BTC, either remained unchanged or slightly decreased. This suggests that during the pandemic period, the degree of efficiency did not undergo as pronounced fluctuations as before the pandemic (Figure A2c,f). This observation implies that perhaps, in times of crisis, the efficiency of the cryptocurrency market could exhibit some degree of resilience. In contrast, for most traditional currencies, the variance experienced a sharp increase, indicating that changes in efficiency levels were more pronounced during the pandemic compared to the pre-pandemic period.

**Figure 7:**
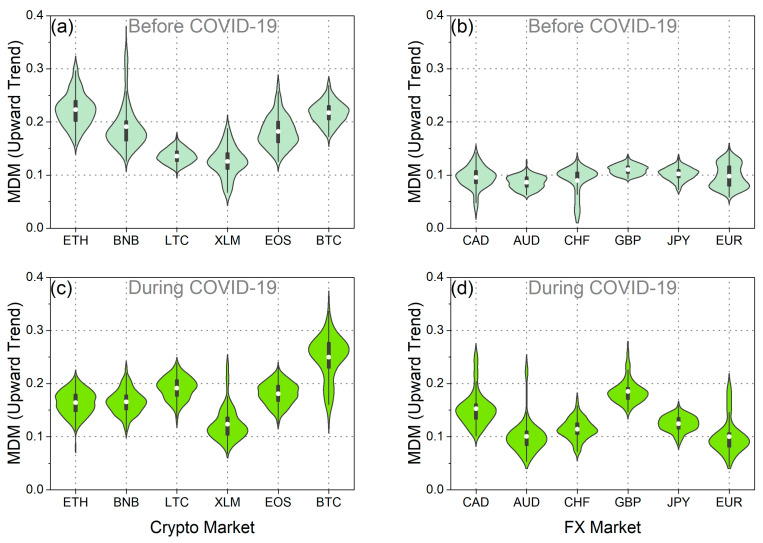
The violin plots of the asymmetric MDM value distribution for the upward market trends of each: (**a**) cryptocurrency in the period before COVID-19, (**b**) traditional currency in the period before COVID-19, (**c**) cryptocurrency during the COVID-19 period, and (**d**) traditional currency during the COVID-19 period.

**Figure 8:**
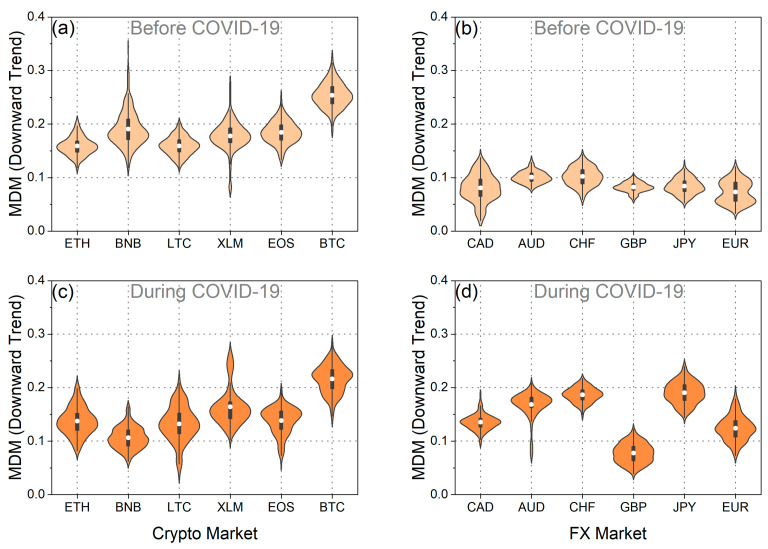
The violin plots of the asymmetric MDM value distribution for the downward market trends of each: (**a**) cryptocurrency in the period before COVID-19, (**b**) traditional currency in the period before COVID-19, (**c**) cryptocurrency during the COVID-19 period, and (**d**) traditional currency during the COVID-19 period.

**Figure A3:**
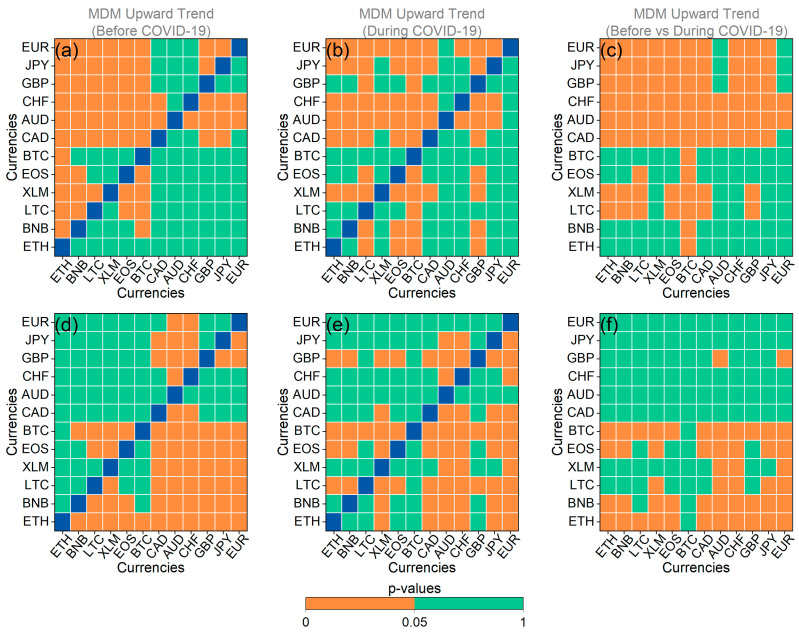
Same as Figure A1, but for the upward market trend.

**Figure A4:**
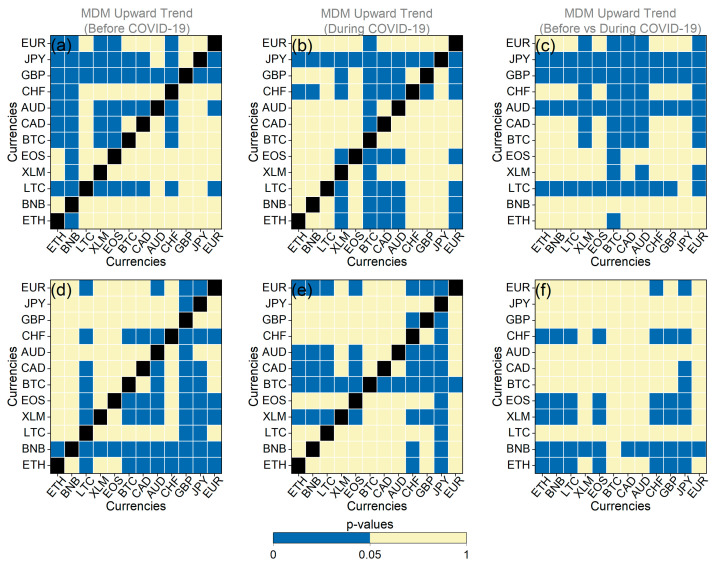
Same as Figure A2, but for the upward market trend.

**Figure A5:**
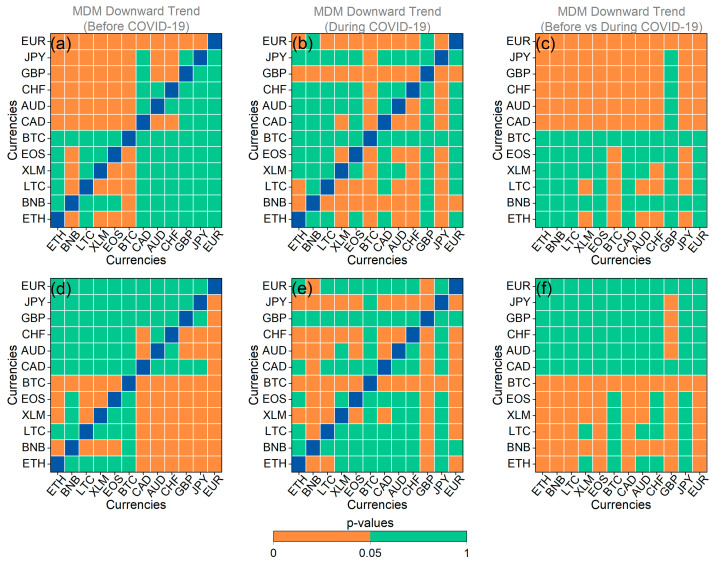
Same as Figure A1, but for the downward market trend.

**Figure A6:**
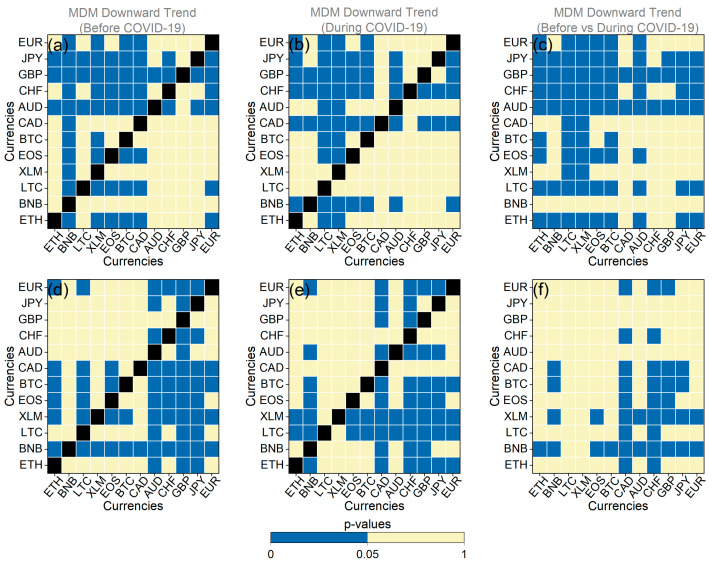
Same as Figure A2, but for the downward market trend.

In Figure 7, Figure 8, Figure A3, Figure A4, Figure A5 and Figure A6, we present the asymmetric MDM violin plots and statistical tests for the additional cryptocurrencies and traditional currencies. Before the pandemic period, we confirm that the results generally align with the overall analysis presented in Figure 6. For both upward and downward market trends, cryptocurrencies exhibited significantly higher mean values of MDM than traditional currencies (Figure 7a,b, Figure 8a,b, Figure A3a,d, and Figure A5a,d). However, during the pandemic period, we uncovered results that deviate from the overall analysis. Specifically, as illustrated in Figure 7d, Figure 8d, Figure A3b,e, and Figure A5b,e, the efficiency of traditional currencies in terms of asymmetric market trends varies more significantly compared to the efficiency in terms of the overall trend (Figure 6d). The asymmetric analysis reveals that after the COVID-19 outbreak, traditional currencies, for the most part, exhibited higher mean values of MDM, higher variances, and varying degrees of efficiency (Figure A3c,f, Figure A4c,f, Figure A5c,f, and Figure A6c,f). Furthermore, the divergence in the degree of efficiency among these currencies became more pronounced. This finding implies that traditional currencies did not exhibit a uniform response after the outbreak; instead, they reacted asymmetrically depending on market trends.

Moreover, our asymmetric study sheds light on how efficiency in cryptocurrencies changed during COVID-19. While the overall and downward market trends analysis (Figure 6a,c and Figure 8a,c) suggests that cryptocurrencies generally displayed lower mean values of MDM (with the exception of BTC in the overall trend analysis), we observed contrasting effects in some cryptocurrencies, especially for the upward market trends (Figure 7a,c and Figure A3c,f). Specifically, when comparing Figure 7a,c, we notice that the efficiency of XLM and EOS remained unaffected by the pandemic during upward market trends, with mean values remaining consistent before and during the pandemic. In contrast, LTC and BTC exhibited higher mean values during the upward market trends, indicating increased inefficiency brought about by COVID-19 during these market trends, but not during downward market trends (Figure A3c,f and Figure A5c,f). Therefore, these cryptocurrencies became more predictable in terms of future returns during the upward market trends. Turning our focus to the variance analysis, we observe that during downward market trends, the variance of MDM values increased in the majority of cryptocurrencies during the pandemic. This suggests that efficiency fluctuations during downward market trends were more pronounced than in the period before COVID-19 (Figure A6c,f). However, during upward market trends, the results were varied, with ETH, BNB, and EOS exhibiting a decrease in variance during the pandemic and BTC and LTC displaying an increase in variance, while the variance of efficiency in XLM did not appear to be significantly affected by the pandemic (Figure A4c,f).

Furthermore, the asymmetric results prompt discussions on how the pandemic affected cryptocurrencies and traditional currencies in terms of market efficiency, highlighting both their similarities and differences. It becomes evident that the gain in efficiency among cryptocurrencies and the loss of efficiency among traditional currencies generally reflect their divergent responses to the pandemic. However, it is crucial to acknowledge that when considering market trends, there are some exceptions to this pattern. Therefore, these empirical findings offer deeper insights into the market behavior during the COVID-19 pandemic and its surrounding period.

**Figure 9:**
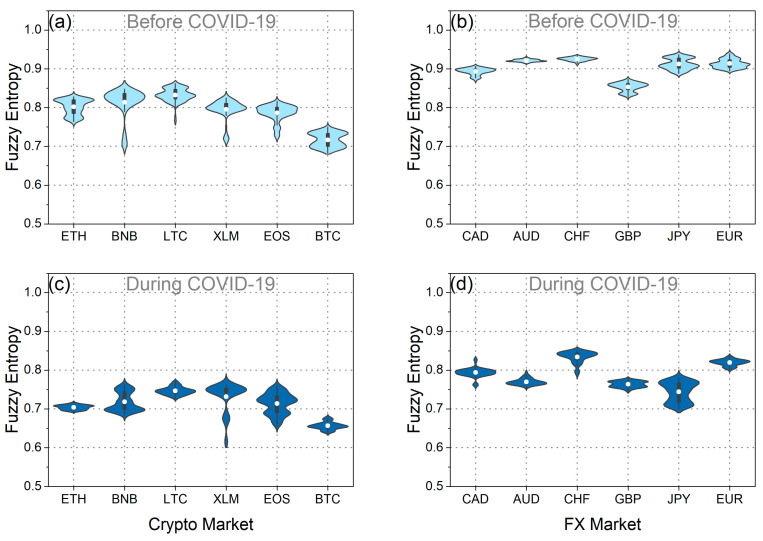
The violin plots of the fuzzy entropy value distribution of each: (**a**) cryptocurrency in the period before COVID-19, (**b**) traditional currency in the period before COVID-19, (**c**) cryptocurrency during the COVID-19 period, and (**d**) traditional currency during the COVID-19 period.

**Figure 10:**
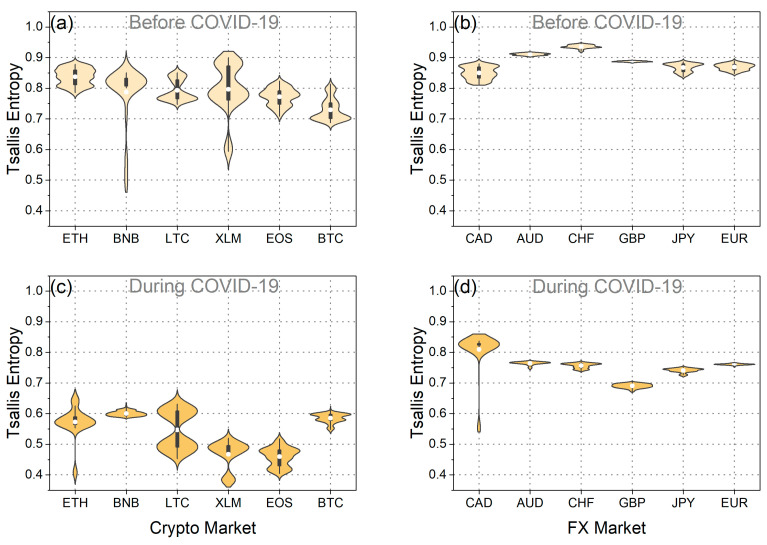
The violin plots of the Tsallis entropy value distribution of each: (**a**) cryptocurrency in the period before COVID-19, (**b**) traditional currency in the period before COVID-19, (**c**) cryptocurrency during the COVID-19 period, and (**d**) traditional currency during the COVID-19 period.

**Figure 11:**
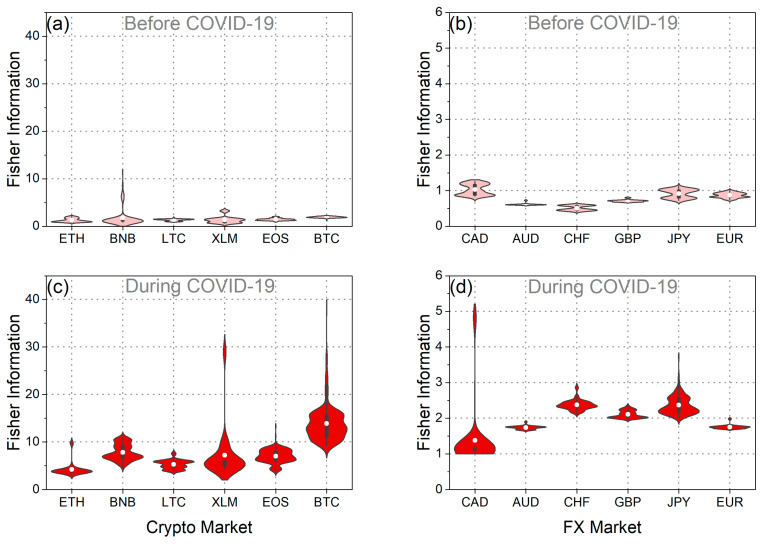
The violin plots of the Fisher information value distribution of each: (**a**) cryptocurrency in the period before COVID-19, (**b**) traditional currency in the period before COVID-19, (**c**) cryptocurrency during the COVID-19 period, and (**d**) traditional currency during the COVID-19 period.

**Figure A7:**
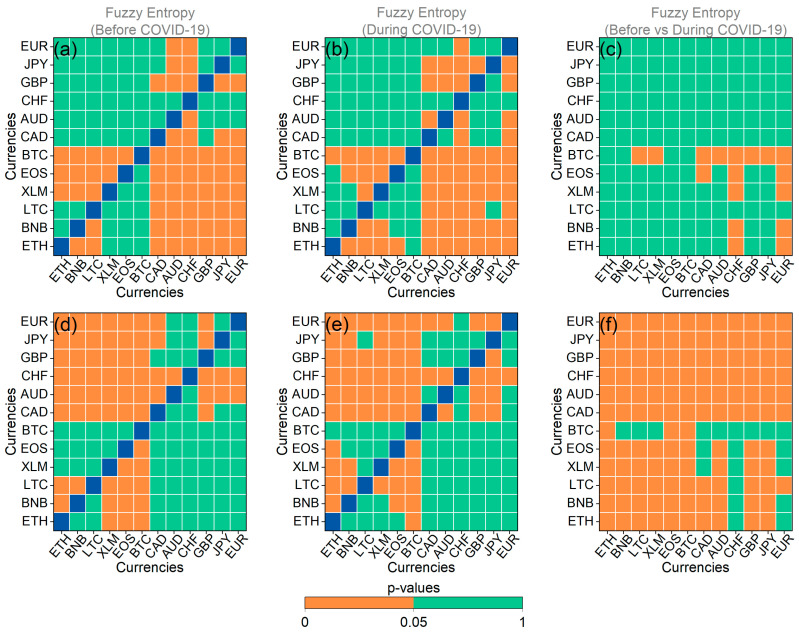
Each heatmap displays one-sided *t*-test probability values. All statistical tests were conducted at a 5% significance level. In the heatmaps: orange color signifies acceptance of the null hypothesis, green color indicates rejection of the null hypothesis, and blue color represents cases where the statistical test is not applicable due to the comparison of identical currencies. Specifically: (**a**) Null hypothesis: the mean value of fuzzy entropy before the pandemic for each currency on the Y-axis is greater than the mean value of fuzzy entropy before the pandemic for each currency on the X-axis. (**b**) Null hypothesis: the mean value of fuzzy entropy during the pandemic for each currency on the Y-axis is greater than the mean value of fuzzy entropy during the pandemic for each currency on the X-axis. (**c**) Null hypothesis: the mean value of fuzzy entropy before the pandemic for each currency on the Y-axis is greater than the mean value of fuzzy entropy during the pandemic for each currency on the X-axis. (**d**) Reverse null hypothesis: the mean value of fuzzy entropy before the pandemic for each currency on the Y-axis is less than the mean value of fuzzy entropy before the pandemic for each currency on the X-axis. (**e**) Reverse null hypothesis: the mean value of fuzzy entropy during the pandemic for each currency on the Y-axis is less than the mean value of fuzzy entropy during the pandemic for each currency on the X-axis. (**f**) Reverse null hypothesis: the mean value of fuzzy entropy before the pandemic for each currency on the Y-axis is less than the mean value of fuzzy entropy during the pandemic for each currency on the X-axis.

Then, we studied fuzzy entropy, Tsallis entropy, and Fisher information for the set of currencies under study. Figure 9a,b, Figure 10a,b, Figure 11a,b, Figure A7a,d, and Figure A9a,d depicting the corresponding statistical tests, illustrate that in the pre-pandemic period, the mean values of fuzzy and Tsallis entropies for cryptocurrencies were lower than those of traditional currencies, indicating less randomness in cryptocurrency returns. Fisher information values for cryptocurrencies were significantly higher than those for traditional currencies during this period, further emphasizing their lower efficiency (Figure 11a,d and Figure A11a,d). This suggests that before the pandemic, the cryptocurrencies we studied exhibited less randomness and were less efficient than traditional currencies. These findings align with the results obtained using the MDM analysis.

**Figure A8:**
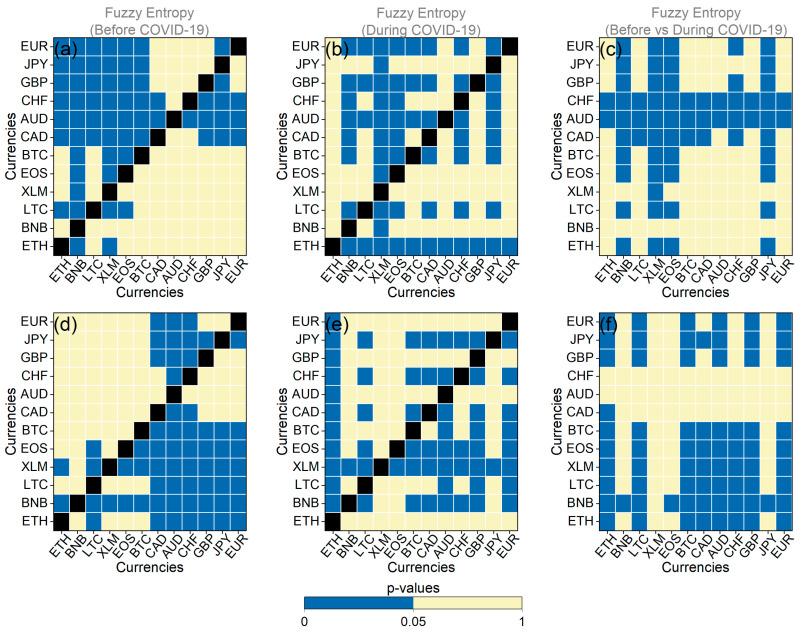
Each heatmap displays F-test probability values. All statistical tests were conducted at a 5% significance level. In the heatmaps: yellow color signifies acceptance of the null hypothesis, blue color indicates rejection of the null hypothesis, and black color represents cases where the statistical test is not applicable due to the comparison of identical currencies. Specifically: (**a**) Null hypothesis: the variance of fuzzy entropy before the pandemic for each currency on the Y-axis is greater than the variance of fuzzy entropy before the pandemic for each currency on the X-axis. (**b**) Null hypothesis: the variance of fuzzy entropy during the pandemic for each currency on the Y-axis is greater than the variance of fuzzy entropy during the pandemic for each currency on the X-axis. (**c**) Null hypothesis: he variance of fuzzy entropy before the pandemic for each currency on the Y-axis is greater than the variance of fuzzy entropy during the pandemic for each currency on the X-axis. (**d**) Reverse null hypothesis: the variance of fuzzy entropy before the pandemic for each currency on the Y-axis is less than the variance of fuzzy entropy before the pandemic for each currency on the X-axis. (**e**) Reverse null hypothesis: the variance of fuzzy entropy during the pandemic for each currency on the Y-axis is less than the variance of fuzzy entropy during the pandemic for each currency on the X-axis. (**f**) Reverse null hypothesis: the variance of fuzzy entropy before the pandemic for each currency on the Y-axis is less than the variance of fuzzy entropy during the pandemic for each currency on the X-axis.

**Figure A9:**
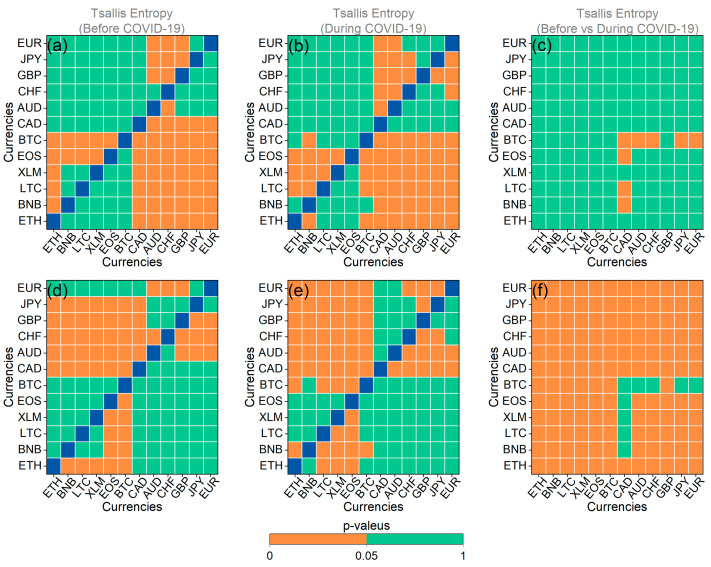
Each heatmap displays one-sided *t*-test probability values. All statistical tests were conducted at a 5% significance level. In the heatmaps: orange color signifies acceptance of the null hypothesis, green color indicates rejection of the null hypothesis, and blue color represents cases where the statistical test is not applicable due to the comparison of identical currencies. Specifically: (**a**) Null hypothesis: the mean value of Tsallis entropy before the pandemic for each currency on the Y-axis is greater than the mean value of Tsallis entropy before the pandemic for each currency on the X-axis. (**b**) Null hypothesis: the mean value of Tsallis entropy during the pandemic for each currency on the Y-axis is greater than the mean value of Tsallis entropy during the pandemic for each currency on the X-axis. (**c**) Null hypothesis: the mean value of Tsallis entropy before the pandemic for each currency on the Y-axis is greater than the mean value of Tsallis entropy during the pandemic for each currency on the X-axis. (**d**) Reverse null hypothesis: the mean value of Tsallis entropy before the pandemic for each currency on the Y-axis is less than the mean value of Tsallis entropy before the pandemic for each currency on the X-axis. (**e**) Reverse null hypothesis: the mean value of Tsallis entropy during the pandemic for each currency on the Y-axis is less than the mean value of Tsallis entropy during the pandemic for each currency on the X-axis. (**f**) Reverse null hypothesis: the mean value of Tsallis entropy before the pandemic for each currency on the Y-axis is less than the mean value of Tsallis entropy during the pandemic for each currency on the X-axis.

**Figure A10:**
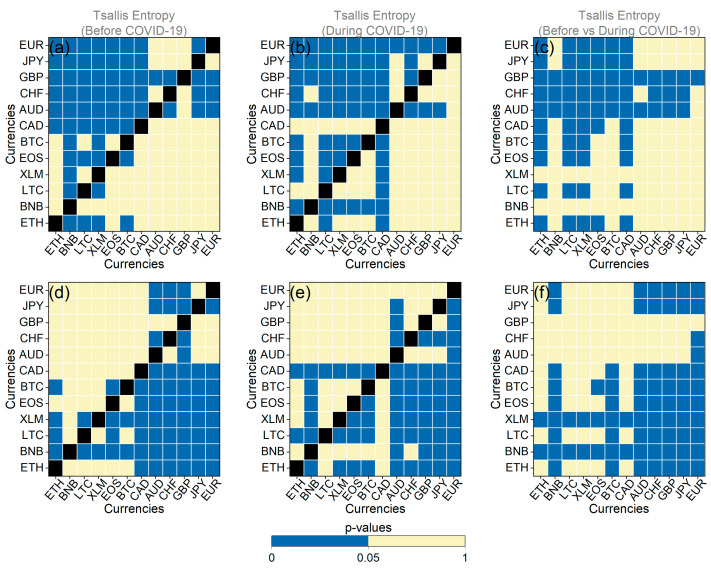
Each heatmap displays F-test probability values. All statistical tests were conducted at a 5% significance level. In the heatmaps: yellow color signifies acceptance of the null hypothesis, blue color indicates rejection of the null hypothesis, and black color represents cases where the statistical test is not applicable due to the comparison of identical currencies. Specifically: (**a**) Null hypothesis: the variance of Tsallis entropy before the pandemic for each currency on the Y-axis is greater than the variance of Tsallis entropy before the pandemic for each currency on the X-axis. (**b**) Null hypothesis: the variance of Tsallis entropy during the pandemic for each currency on the Y-axis is greater than the variance of Tsallis entropy during the pandemic for each currency on the X-axis. (**c**) Null hypothesis: the variance of Tsallis entropy before the pandemic for each currency on the Y-axis is greater than the variance of Tsallis entropy during the pandemic for each currency on the X-axis. (**d**) Reverse null hypothesis: the variance of Tsallis entropy before the pandemic for each currency on the Y-axis is less than the variance of Tsallis entropy before the pandemic for each currency on the X-axis. (**e**) Reverse null hypothesis: the variance of Tsallis entropy during the pandemic for each currency on the Y-axis is less than the variance of Tsallis entropy during the pandemic for each currency on the X-axis. (**f**) Reverse null hypothesis: the variance of Tsallis entropy before the pandemic for each currency on the Y-axis is less than the variance of Tsallis entropy during the pandemic for each currency on the X-axis.

**Figure A11:**
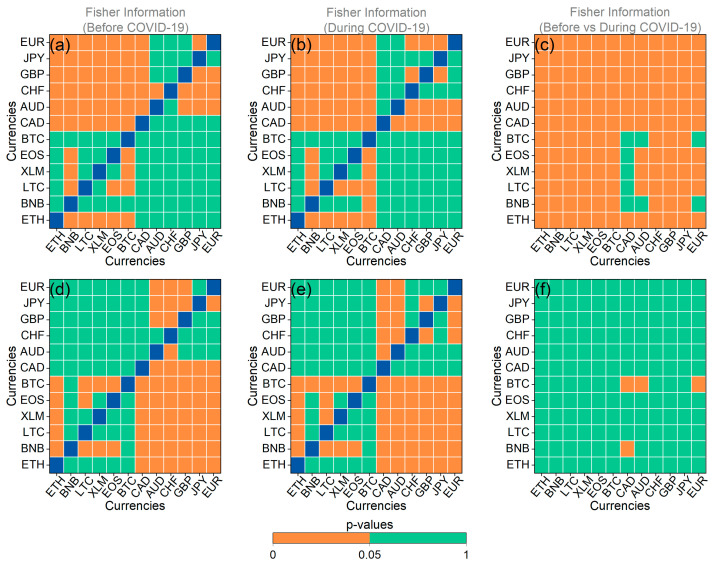
Each heatmap displays one-sided *t*-test probability values. All statistical tests were conducted at a 5% significance level. In the heatmaps: orange color signifies acceptance of the null hypothesis, green color indicates rejection of the null hypothesis, and blue color represents cases where the statistical test is not applicable due to the comparison of identical currencies. Specifically: (**a**) Null hypothesis: the mean value of Fisher information before the pandemic for each currency on the Y-axis is greater than the mean value of Fisher information before the pandemic for each currency on the X-axis. (**b**) Null hypothesis: the mean value of Fisher information during the pandemic for each currency on the Y-axis is greater than the mean value of Fisher information during the pandemic for each currency on the X-axis. (**c**) Null hypothesis: the mean value of Fisher information before the pandemic for each currency on the Y-axis is greater than the mean value of Fisher information during the pandemic for each currency on the X-axis. (**d**) Reverse null hypothesis: the mean value of Fisher information before the pandemic for each currency on the Y-axis is less than the mean value of Fisher information before the pandemic for each currency on the X-axis. (**e**) Reverse null hypothesis: the mean value of Fisher information during the pandemic for each currency on the Y-axis is less than the mean value of Fisher information during the pandemic for each currency on the X-axis. (**f**) Reverse null hypothesis: the mean value of Fisher information before the pandemic for each currency on the Y-axis is less than the mean value of Fisher information during the pandemic for each currency on the X-axis.

**Figure A12:**
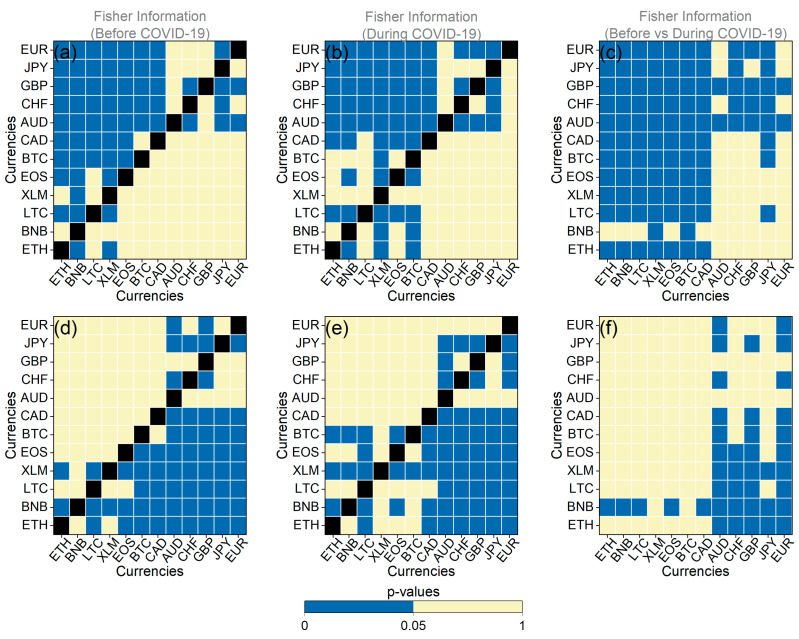
Each heatmap displays F-test probability values. All statistical tests were conducted at a 5% significance level. In the heatmaps: yellow color signifies acceptance of the null hypothesis, blue color indicates rejection of the null hypothesis, and black color represents cases where the statistical test is not applicable due to the comparison of identical currencies. Specifically: (**a**) Null hypothesis: the variance of Fisher information before the pandemic for each currency on the Y-axis is greater than the variance of Fisher information before the pandemic for each currency on the X-axis. (**b**) Null hypothesis: the variance of Fisher information during the pandemic for each currency on the Y-axis is greater than the variance of Fisher information during the pandemic for each currency on the X-axis. (**c**) Null hypothesis: the variance of Fisher information before the pandemic for each currency on the Y-axis is greater than the variance of Fisher information during the pandemic for each currency on the X-axis. (**d**) Reverse null hypothesis: the variance of Fisher information before the pandemic for each currency on the Y-axis is less than the variance of Fisher information before the pandemic for each currency on the X-axis. (**e**) Reverse null hypothesis: the variance of Fisher information during the pandemic for each currency on the Y-axis is less than the variance of Fisher information during the pandemic for each currency on the X-axis. (**f**) Reverse null hypothesis: the variance of Fisher information before the pandemic for each currency on the Y-axis is less than the variance of Fisher information during the pandemic for each currency on the X-axis.

When we examine the pandemic period, it becomes evident that both cryptocurrencies and traditional currencies experienced a decrease in efficiency. This is apparent from the decline in mean entropy values and the increase in mean Fisher information values compared to the pre-pandemic period (Figure A7c,f, Figure A9c,f, and Figure A11c,f). These results align seamlessly with the findings obtained using the MDM method for traditional currencies. Conversely, for the majority of cryptocurrencies, the results of entropies and Fisher information do not concur with the outcomes of the MDM method. The only exception within the realm of cryptocurrencies is BTC, where all methods converge on the conclusion that BTC became less efficient during the pandemic. Interesting observations also arise from the analysis of the variance in the distributions of these three measures. In general, we observe that, in the majority of the traditional currencies we studied, the fluctuations in the values of all measures increased during the pandemic (Figure A8c,f, Figure A10c,f, and Figure A12c,f). This suggests that the outbreak of the pandemic caused more widespread instability in the efficiency levels of the forex markets. Conversely, for the majority of cryptocurrencies, it appears that the variance of the distributions of the measures we studied decreased. However, the exception is the value distribution of Fisher information, where for the majority of traditional currencies and cryptocurrencies, the variance has increased.

## 5. Conclusions

The COVID-19 pandemic caused an unimagined impact on the world economy, business, social development, and financial markets. The great uncertainty caused by the pandemic among investors led to fear and extreme volatility in the financial markets. As expected, the impact could not leave the cryptocurrency market, which is a new and emerging market, or the forex market, which is the largest market in the world, unaffected. Motivated by the significant role these markets play in the modern financial system and considering the substantial impact of COVID-19 on them, we conducted an in-depth comparative analysis to explore the effects of COVID-19 on the weak-form efficiency of both the cryptocurrency and forex markets. Specifically, we examined the temporal evolution of the asymmetric MDM method, fuzzy entropy, Tsallis entropy, and Fisher information. Additionally, we followed an alternative approach to analyze the temporal evolution of these four measures by studying their value distributions before and during COVID-19. In this way, significant information was revealed regarding the change in the weak-form efficiency of each currency.

The main results obtained using our analysis can be summarized as follows: using the analysis of MDM (in overall market trend), fuzzy entropy, Tsallis entropy, and Fisher information, the following observations were made: (i) Both before and during the pandemic, BTC was found to be less efficient than EUR. Consequently, it can be concluded that BTC was more predictable than EUR. Also, after the onset of the pandemic, the mean values of MDM and Fisher information increased compared to the previous period, while the mean values of fuzzy entropy and Tsallis entropy decreased for both BTC and EUR. This observation indicates that the pandemic led to decreased efficiency in both currencies. This finding aligns with the results of a recent study by Zitis et al. [20], in which the returns of both BTC and EUR exhibited reduced complexity and an increase in multifractality. These findings also support the increase in inefficiency and the fact that assets have become less random. (ii) The variance of MDM value for both BTC and EUR increased during the pandemic. This suggests that the changes in their efficiency levels were more pronounced during the pandemic than before the pandemic. When examining the distributions of entropies, as well as the Fisher information, it becomes evident that in the case of EUR, the variance has decreased. Conversely, in the case of BTC, the variance of the entropy values decreased while the variance of the Fisher information values increased. Therefore, considering all the measures we used collectively, it appears that we cannot arrive at a definitive conclusion regarding whether the pandemic has changed the volatility in the efficiency levels of the two currencies more than before. (iii) In the period before the pandemic, the cryptocurrencies studied were, on the whole, less efficient than the traditional currencies under examination. This conclusion is supported by all the methods we applied. An interesting insight that emerges from the overall trend analysis of MDM is that cryptocurrencies appear to exhibit varying levels of efficiency, in contrast to traditional currencies, which demonstrate more uniform levels of efficiency. Therefore, we can conclude that cryptocurrencies were more predictable than traditional currencies, but each cryptocurrency displayed different levels of predictability. The observation of inefficiency in cryptocurrencies aligns with existing literature, as several academic studies provide evidence of the inefficiency of BTC and other cryptocurrencies [67]. (iv) During the pandemic period, the overall trend analysis of MDM reveals that the majority of studied cryptocurrencies exhibited increased efficiency compared to the preceding period. This finding is partially consistent with the results of Mnif et al. [25], who found that the efficiency of ETH, LTC, BNB, and BTC increased during the pandemic compared to the previous period. In contrast, all the traditional currencies studied became less effective. Similar conclusions were reached by Aslam et al. [26], who found, among other things, that during the COVID-19 pandemic, the efficiency of the foreign exchange markets decreased. Furthermore, the overall trend analysis of MDM reveals that, while before the pandemic, cryptocurrencies appeared to exhibit varying levels of efficiency, with traditional currencies showing similar levels of efficiency, after the outbreak of the pandemic, it seems that traditional currencies displayed varying levels of efficiency, while the majority of cryptocurrencies demonstrated similar levels of efficiency. Upon examining the results derived from entropies and Fisher information, it is evident that both cryptocurrencies and traditional currencies experienced a decrease in efficiency. These results align seamlessly with the findings obtained using the MDM method for traditional currencies. Conversely, for the majority of cryptocurrencies, the results of entropies and Fisher information do not concur with the outcomes of the MDM method. The only exception within the realm of cryptocurrencies is BTC, where all methods converge on the conclusion that BTC became less efficient during the pandemic. (v) During the pandemic, BTC was the only cryptocurrency that exhibited a behavior similar to that of the traditional currencies, as they became less efficient. The decrease in BTC efficiency after the outbreak of the pandemic is consistent with the results of Kakinaka and Umeno [28], who found that on the short-term horizon, the efficiency of BTC decreased during the pandemic period compared to the previous period. (vi) Analyzing the variance of the distributions of all the measures we studied, it is revealed that in the case of the majority of traditional currencies, the variance has increased. Consequently, it can be inferred that changes in their efficiency levels were more pronounced during the pandemic than before the pandemic. Conversely, for the majority of cryptocurrencies, it appears that the variance of the distributions of the measures we studied decreased. One exception is the price distribution of Fisher information, where for both the majority of traditional currencies and the majority of cryptocurrencies, the variance has increased.

Using asymmetric analysis, it is revealed that: (i) Before and during the pandemic, both for the upward and downward market trends, the mean of the MDM values of BTC was higher than the corresponding mean of the MDM values of EUR. This supports the findings that EUR was more efficient than BTC under any market trend. (ii) During the pandemic period, BTC became more efficient during falling markets, while in all the other cases, market inefficiency increased. During the same period, EUR became more inefficient during falling markets, while on the contrary, for the upward market trends, the mean value of MDM was kept at the same level both before and during the pandemic. These findings show that the predictability of future returns of EUR was higher during falling markets after the outbreak of the pandemic compared to the pre-pandemic period. In contrast, the predictability of future returns of BTC was lower during falling markets after the outbreak of the pandemic compared to the pre-pandemic period. (iii) After the COVID-19 outbreak, the studied traditional currencies in their majority appear to have a higher mean, a higher variance, and varying degrees of efficiency. Also, the divergence in the degree of efficiency among these currencies is more pronounced. This finding implies that traditional currencies after the outbreak did not show a common behavior, and they reacted asymmetrically depending on market trends. (iv) During the pandemic period, some of the studied cryptocurrencies showed opposite results in different market trends. Therefore, it is concluded that the predictability of future returns of some cryptocurrencies varies according to market trends. (v) During downward market trends, the variance of MDM values increased during the pandemic in the majority of the studied cryptocurrencies. This fact suggests that efficiency fluctuations during downward market trends were sharper than in the period before COVID-19. On the other hand, during upward market trends, the results are varied.

In summary, the results of our study reveal that the pandemic had a notable impact on the weak-form efficiency of the currencies under examination. These findings are consistent with Lo’s adaptive market hypothesis [4], which suggests that market efficiency changes over time. Consequently, it can be inferred that investors adapted their investment strategies in response to the changing financial landscape, particularly following the pandemic’s outbreak. Furthermore, our analysis indicates that the weak-form efficiency levels of the majority of traditional currencies experienced more pronounced changes after the pandemic outbreak, whereas cryptocurrencies displayed a contrasting reaction. This observation implies that, perhaps, the cryptocurrency market may exhibit a degree of resilience during times of crisis. Finally, another interesting finding that emerged from our analysis is the behavior of BTC. Specifically, during the pandemic, BTC was the only cryptocurrency that exhibited behavior similar to that of traditional currencies as they became less efficient. This fact may provide some evidence of BTC’s maturity. As a result, the insights provided in our article could prove valuable for investors, portfolio managers, and policymakers. Investors and portfolio managers should consider market trends—both upward and downward—when forecasting currency prices, as the ability to outperform these markets and achieve abnormal returns can vary based on market conditions. Additionally, they can capitalize on periods of market inefficiency to anticipate future returns and generate profits. Because market inefficiency suggests the existence of market imperfections and behavioral biases, policymakers may consider implementing new reforms aimed at enhancing the efficiency of the cryptocurrency market, in particular.

## Figures and Tables

**Table 1:** Reported probability values using one-sided *t*-test to compare MDM mean values for BTC and EUR. All statistical tests were performed at 5% statistical significance level.

Statistical Tests			
Asymmetric MDM			
	Overall Trend	Upward Trend	Downward Trend
Null Hypothesis	*p*-Value	*p*-Value	*p*-Value
MDM Mean (BTC) Before COVID-19 > MDM Mean (EUR) Before COVID-19	1.0000	1.0000	1.0000
MDM Mean (BTC) Before COVID-19 < MDM Mean (EUR) Before COVID-19	0.0000	0.0000	0.0000
MDM Mean (BTC) During COVID-19 > MDM Mean (EUR) During COVID-19	1.0000	1.0000	1.0000
MDM Mean (BTC) During COVID-19 < MDM Mean (EUR) During COVID-19	0.0000	0.0000	0.0000
MDM Mean (BTC) Before COVID-19 > MDM Mean (BTC) During COVID-19	0.0000	0.0000	1.0000
MDM Mean (BTC) Before COVID-19 < MDM Mean (BTC) During COVID-19	1.0000	1.0000	0.0000
MDM Mean (EUR) Before COVID-19 > MDM Mean (EUR) During COVID-19	0.0000	0.2319	0.0000
MDM Mean (EUR) Before COVID-19 < MDM Mean (EUR) During COVID-19	1.0000	0.7681	1.0000
MDM Mean (BTC) Before COVID-19 > MDM Mean (EUR) During COVID-19	1.0000	1.0000	1.0000
MDM Mean (BTC) Before COVID-19 < MDM Mean (EUR) During COVID-19	0.0000	0.0000	0.0000
MDM Mean (EUR) Before COVID-19 > MDM Mean (BTC) During COVID-19	0.0000	0.0000	0.0000
MDM Mean (EUR) Before COVID-19 < MDM Mean (BTC) During COVID-19	1.0000	1.0000	1.0000

**Table 2:** Reported probability values using one-sided F-test to compare MDM variance values for BTC and EUR. All statistical tests were performed at a 5% statistical significance level.

Statistical Tests			
Asymmetric MDM			
	Overall Trend	Upward Trend	Downward Trend
Null Hypothesis	*p*-Value	*p*-Value	*p*-Value
MDM Variance (BTC) Before COVID-19 > MDM Variance (EUR) Before COVID-19	0.9977	0.2204	0.9762
MDM Variance (BTC) Before COVID-19 < MDM Variance (EUR) Before COVID-19	0.0023	0.7796	0.0238
MDM Variance (BTC) During COVID-19 > MDM Variance (EUR) During COVID-19	0.9209	0.9999	0.9715
MDM Variance (BTC) During COVID-19 < MDM Variance (EUR) During COVID-19	0.0791	0.0001	0.0285
MDM Variance (BTC) Before COVID-19 > MDM Variance (BTC) During COVID-19	0.0000	0.0000	0.0335
MDM Variance (BTC) Before COVID-19 < MDM Variance (BTC) During COVID-19	1.0000	1.0000	0.9665
MDM Variance (EUR) Before COVID-19 > MDM Variance (EUR) During COVID-19	0.0000	0.0000	0.0536
MDM Variance (EUR) Before COVID-19 < MDM Variance (EUR) During COVID-19	1.0000	1.0000	0.9464
MDM Variance (BTC) Before COVID-19 > MDM Variance (EUR) During COVID-19	0.0000	0.0000	0.5991
MDM Variance (BTC) Before COVID-19 < MDM Variance (EUR) During COVID-19	1.0000	1.0000	0.4009
MDM Variance (EUR) Before COVID-19 > MDM Variance (BTC) During COVID-19	0.0000	0.0000	0.0002
MDM Variance (EUR) Before COVID-19 < MDM Variance (BTC) During COVID-19	1.0000	1.0000	0.9998

**Table 3:** Reported probability values using one-sided *t*-test to compare complexity measures’ (fuzzy and Tsallis entropies, and Fisher information) mean values for BTC and EUR. All statistical tests were performed at a 5% statistical significance level.

Statistical Tests			
Entropies and Information			
	Fuzzy Entropy	Tsallis Entropy	Fisher Information
Null Hypothesis	*p*-Value	*p*-Value	*p*-Value
Ent./Info. Mean (BTC) Before COVID-19 > Ent./Info. Mean (EUR) Before COVID-19	0.0000	0.0000	1.0000
Ent./Info. Mean (BTC) Before COVID-19 < Ent./Info. Mean (EUR) Before COVID-19	1.0000	1.0000	0.0000
Ent./Info. Mean (BTC) During COVID-19 > Ent./Info. Mean (EUR) During COVID-19	0.0000	0.0000	1.0000
Ent./Info. Mean (BTC) During COVID-19 < Ent./Info. Mean (EUR) During COVID-19	1.0000	1.0000	0.0000
Ent./Info. Mean (BTC) Before COVID-19 > Ent./Info. Mean (BTC) During COVID-19	1.0000	1.0000	0.0000
Ent./Info. Mean (BTC) Before COVID-19 < Ent./Info. Mean (BTC) During COVID-19	0.0000	0.0000	1.0000
Ent./Info. Mean (EUR) Before COVID-19 > Ent./Info. Mean (EUR) During COVID-19	1.0000	1.0000	0.0000
Ent./Info. Mean (EUR) Before COVID-19 < Ent./Info. Mean (EUR) During COVID-19	0.0000	0.0000	1.0000
Ent./Info. Mean (BTC) Before COVID-19 > Ent./Info. Mean (EUR) During COVID-19	0.0000	0.0000	1.0000
Ent./Info. Mean (BTC) Before COVID-19 < Ent./Info. Mean (EUR) During COVID-19	1.0000	1.0000	0.0000
Ent./Info. Mean (EUR) Before COVID-19 > Ent./Info. Mean (BTC) During COVID-19	1.0000	1.0000	0.0000
Ent./Info. Mean (EUR) Before COVID-19 < Ent./Info. Mean (BTC) During COVID-19	0.0000	0.0000	1.0000

**Table 4:** Reported probability values using one-sided F-test to compare complexity measures’ (fuzzy and Tsallis entropies, and Fisher information) variance values for BTC and EUR. All statistical tests were performed at a 5% statistical significance level.

Statistical Tests			
Entropies and Information			
	Fuzzy Entropy	Tsallis Entropy	Fisher Information
Null Hypothesis	*p*-Value	*p*-Value	*p*-Value
Ent./Info. Variance (BTC) Before COVID-19 > Ent./Info. Variance (EUR) Before COVID-19	1.0000	1.0000	1.0000
Ent./Info. Variance (BTC) Before COVID-19 < Ent./Info. Variance (EUR) Before COVID-19	0.0000	0.0000	0.0000
Ent./Info. Variance (BTC) During COVID-19 > Ent./Info. Variance (EUR) During COVID-19	0.9961	1.0000	1.0000
Ent./Info. Variance (BTC) During COVID-19 < Ent./Info. Variance (EUR) During COVID-19	0.0039	0.0000	0.0000
Ent./Info. Variance (BTC) Before COVID-19 > Ent./Info. Variance (BTC) During COVID-19	1.0000	1.0000	0.0000
Ent./Info. Variance (BTC) Before COVID-19 < Ent./Info. Variance (BTC) During COVID-19	0.0000	0.0000	1.0000
Ent./Info. Variance (EUR) Before COVID-19 > Ent./Info. Variance (EUR) During COVID-19	1.0000	1.0000	0.9991
Ent./Info. Variance (EUR) Before COVID-19 < Ent./Info. Variance (EUR) During COVID-19	0.0000	0.0000	0.0009
Ent./Info. Variance (BTC) Before COVID-19 > Ent./Info. Variance (EUR) During COVID-19	1.0000	1.0000	1.0000
Ent./Info. Variance (BTC) Before COVID-19 < Ent./Info. Variance (EUR) During COVID-19	0.0000	0.0000	0.0000
Ent./Info. Variance (EUR) Before COVID-19 > Ent./Info. Variance (BTC) During COVID-19	0.9957	0.0000	0.0000
Ent./Info. Variance (EUR) Before COVID-19 < Ent./Info. Variance (BTC) During COVID-19	0.0043	1.0000	1.0000

## Data Availability

All financial time series used in this article are publicly available from Yahoo Finance (http://finance.yahoo.com/, accessed on 1 October 2023).
